# The Fine Art of Writing a Message: RNA Metabolism in the Shaping and Remodeling of the Nervous System

**DOI:** 10.3389/fnmol.2021.755686

**Published:** 2021-11-30

**Authors:** María Landínez-Macías, Olivier Urwyler

**Affiliations:** ^1^Department of Molecular Life Sciences, University of Zurich, Zurich, Switzerland; ^2^Molecular Life Sciences Program, Life Science Zurich Graduate School, University of Zurich and Eidgenössische Technische Hochschule (ETH) Zurich, Zurich, Switzerland; ^3^Neuroscience Center Zurich (ZNZ), University of Zurich, Zurich, Switzerland

**Keywords:** RNA-binding proteins, neuronal wiring, RNA metabolism, Musashi, neuronal development, neurological diseases, post-transcriptional control of gene expression

## Abstract

Neuronal morphogenesis, integration into circuits, and remodeling of synaptic connections occur in temporally and spatially defined steps. Accordingly, the expression of proteins and specific protein isoforms that contribute to these processes must be controlled quantitatively in time and space. A wide variety of post-transcriptional regulatory mechanisms, which act on pre-mRNA and mRNA molecules contribute to this control. They are thereby critically involved in physiological and pathophysiological nervous system development, function, and maintenance. Here, we review recent findings on how mRNA metabolism contributes to neuronal development, from neural stem cell maintenance to synapse specification, with a particular focus on axon growth, guidance, branching, and synapse formation. We emphasize the role of RNA-binding proteins, and highlight their emerging roles in the poorly understood molecular processes of RNA editing, alternative polyadenylation, and temporal control of splicing, while also discussing alternative splicing, RNA localization, and local translation. We illustrate with the example of the evolutionary conserved Musashi protein family how individual RNA-binding proteins are, on the one hand, acting in different processes of RNA metabolism, and, on the other hand, impacting multiple steps in neuronal development and circuit formation. Finally, we provide links to diseases that have been associated with the malfunction of RNA-binding proteins and disrupted post-transcriptional regulation.

## 1. Introduction

Developmental assembly of neural circuits occurs through precisely orchestrated cellular events for the specification, differentiation, and morphogenesis of neurons. Neurons typically develop an elaborate dendritic tree and an elongated axon to reach their target area(s) and their specific synaptic partners. Through axon branch formation, neurons can, on the one hand, project to distinct target areas, and, on the other hand, increase the number of presynapses that they can form at a particular location (i.e., the local synaptogenic potential). Dendritic, axonal, and synaptic morphogenesis depend on the ability of the neuron to integrate and to appropriately respond to intrinsic and extrinsic cues. Different neuronal types can use the same set of proteins to respond to these cues. Moreover, these proteins can be reused by a given neuron at different developmental stages and throughout distinct steps in circuit assembly, sometimes with different or even opposing outcomes on neuronal morphogenesis. The response of a neuron thereby depends on the combination of proteins that it expresses at a given time and place. For example, depending on the specific co-receptor that it binds to, a cell-surface receptor can elicit either an attractive or a repulsive cellular response (Dalpé et al., 2004; Chauvet et al., 2007). Proper circuit assembly therefore highly depends on precisely regulated temporal changes of the global cellular proteome, but also on the spatially and temporally controlled composition of local proteomes in dendrites, axons and at synapses. Beyond transcriptional control of gene expression, post-transcriptional mechanisms confer multiple additional layers and means of regulation for achieving protein synthesis at the right time and place in developing neurons. We refer to these mechanisms, which are introduced below, as “RNA metabolism.” RNA-binding proteins (RBPs) are key regulators of RNA metabolism. RBPs are, therefore, critically involved in the expansion of proteome diversity and of proteome function in neurons, and in the rapid and localized control of neuronal gene expression. In turn, these processes are essential for coordinating axon and dendrite growth, guidance, targeting, and synapse formation. In this review, we will discuss how RNA metabolism and its control by RBPs guide key steps of neuronal wiring, with a special focus on axon and synapse development. Given their essential functions in neural circuit assembly, it is not surprising that mutations in RBPs have been associated with neurodevelopmental disorders in humans. We will exemplify this for several RBPs in the last section of this article.

**Figure 1:**
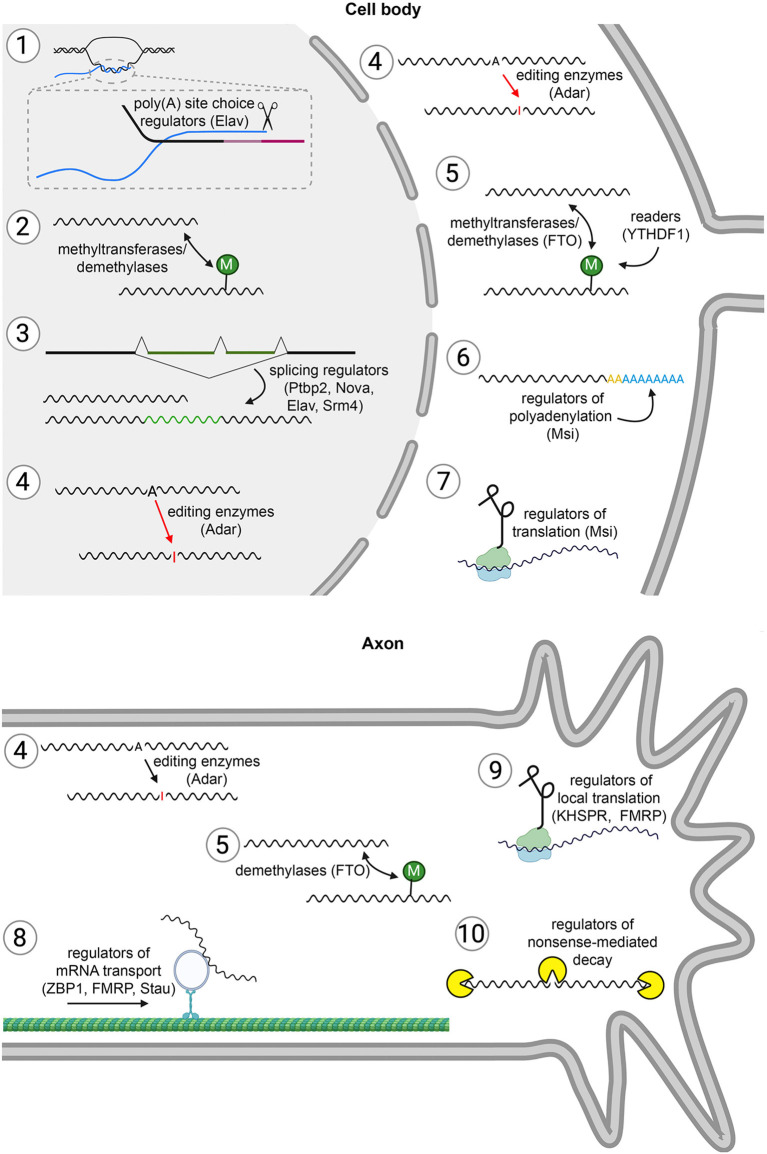
Different steps of mRNA metabolism. (1) Alternative cleavage and polyadenylation. The choice between alternative polyadenylation signals diversifies 3′UTRs and thereby determines, which regulatory motifs are included in an mRNA molecule. Thus, this process expands the potential for post-transcriptional regulation of gene expression. As in the rest of the figure, an example of an RNA-binding protein that controls the process, and which we discuss in this article, is indicated in brackets. Blue: mRNA. Shades of pink: alternative 3′UTRs encoded in the DNA. (2) RNA modification. Nucleotides are modified for example by methyltransferases and demethylases. RNA modification can occur both in the nucleus and in the cytoplasm. This impacts on splicing, translation, and stability of an mRNA. (3) Alternative splicing (AS). Regulation of gene expression by AS is a means to increase proteome diversity, and also to include or exclude regulatory elements that for example can provide temporal or spatial control of expression. (4) RNA editing. The coding and regulatory regions in an mRNA molecule can be edited in the nucleus or in the cytoplasm, for example by conversion of adenosines (A) into inosines (I). (5) Dynamic RNA modifications. In the cell body and axonal cytoplasm, modifications can be added or removed from mRNA molecules. RBPs that recognize the modifications (“readers”) can then for example modulate translation. (6) Polyadenylation. This dynamic process can occur in the nucleus [yellow poly(A) tails] and in the cytoplasm [blue poly(A) tails]. Polyadenylation is a means to regulate translation and mRNA stability. (7) Translation. In neurons, protein synthesis is heavily regulated to provide temporal and spatial control of proteome composition during development. (8) Localization. mRNA molecules can be transported to axon terminals, and (9) locally translated. (10) Degradation. Different pathways, such as nonsense-mediated decay, degrade mRNAs after translation.

RNA metabolism encompasses all the controllable molecular processes that determine the properties of an RNA during its life cycle, from synthesis to degradation (Figure 1). As we are focusing on post-transcriptional regulation of RNAs, we will not discuss RNA biogenesis (i.e., transcription) here. Moreover, we will limit our review to the metabolism of protein-coding RNAs, i.e., messenger RNAs (mRNAs). After transcription, maturation of a pre-mRNA to an mRNA occurs through splicing, 5′ end capping, and 3′ end polyadenylation. The process of splicing allows for a multitude of ways to control the function of an mRNA. First, alternative splicing (AS) of coding exons generates different protein isoforms from a single gene. Such expansion of proteome diversity through AS is prominent for example for neuronal cell-surface adhesion and signaling receptors such as the vertebrate Neurexins and the invertebrate Dscam1 protein (Ushkaryov et al., 1992; Ushkaryov and Südhof, 1993; Schmucker et al., 2000). Second, AS of untranslated regions (UTRs) can modify, which cis-regulatory elements are included in an mRNA molecule. Third, regulated splicing can contribute to the temporal control of gene expression (Mauger et al., 2016). Polyadenylation, i.e., the addition of a poly(A) tail at the 3′ end of the mRNA, also impacts mRNA function in different ways. On the one hand, the choice of the position of poly(A) tail addition determines the length of the 3′-UTR, and thus the inclusion of specific cis-regulatory elements. On the other hand, the length of the poly(A) tail itself contributes to the control of mRNA stability and to its translation rate. After nuclear export, cytoplasmic polyadenylation can lead to further elongation of the poly(A) tail, while cytoplasmic deadenylases are catalyzing poly(A) tail shortening (Wiederhold and Passmore, 2010). Both the 5′ end cap structure and the 3′ end poly(A) tail are important for the stability of the mRNA, and their removal leads to rapid mRNA degradation in most cell types. At the pre-mRNA stage, or after mRNA maturation, coding and regulatory sequences of the mRNA can be altered by post-transcriptional editing through RNA editing enzymes (Savva et al., 2012; Lerner et al., 2018), and such editing can also affect mRNA structure. RNA editing enzymes belong either to the “adenosine deaminases that act on RNA” (ADAR) family or to the “Apolipoprotein B mRNA editing enzyme, catalytic polypeptide-like” (APOBEC) family, and they catalyze the deamination of adenosine to inosine (decoded as guanosine) and cytosine to uracil, respectively. Moreover, RNA modification, of which the most prominent example is adenosine methylation at position *N*^6^ (m^6^A) (Dominissini et al., 2012; Meyer et al., 2012), impinges on mRNA metabolism, including splicing, translation, and stability (Frye et al., 2018). A further aspect that we include here as part of RNA metabolism is the localization of mRNA molecules to specific subcellular compartments, either through passive diffusion and trapping at a particular location, or through active transport by motor proteins along microtubules or the actin cytoskeleton. Such RNA localization can target the synthesis of specific proteins to defined subcellular locations through local translation. Finally, the output of a given mRNA molecule is determined by its translational rate, which is highly regulated by different means and through different molecular players. We will present in the following sections different examples for these various facets of mRNA metabolism, and how they individually, or in combination, impact neuronal development. Given the breadth of identified mechanisms and molecules, we are thereby focusing on chosen examples rather than aiming at providing a comprehensive review of the field.

## 2. Roles of RNA Metabolism in Neuronal Wiring and Remodeling

### 2.1. Alternative Cleavage and Polyadenylation

During transcription, the recognition of a polyadenylation signal (PAS) triggers the downstream cleavage of the nascent transcript, and thus the release of a pre-mRNA molecule. Concomitantly, the PAS induces nuclear polyadenylation of the pre-mRNA. The recognition and usage of alternative transcript termination sites/PASs, known as alternative cleavage and polyadenylation (APA), diversifies the 3′UTRs of mRNA isoforms and their regulatory potential. Indeed, changing the 3′UTR length directs the exclusion or inclusion of more downstream structural and cis-regulatory elements. APA patterns are tissue-specific, and even mRNAs that are ubiquitously expressed in many tissues have alternative 3′UTRs that are used at different ratios in each tissue (Lianoglou et al., 2013). Overall, neuronal tissues are biased toward expressing isoforms with longer 3′UTRs (Miura et al., 2013; Guvenek and Tian, 2018). For instance, during embryogenesis in *Drosophila*, some mRNAs in neuronal tissues can have 3′UTRs up to 20-fold longer than the 3′UTRs of the same mRNAs in other tissues (Hilgers et al., 2011). Elongation of the 3′UTR starts early in the development of the nervous system, with neuronal stem cells already having longer 3′UTRs, which is required for correct neuronal differentiation (Grassi et al., 2018). During the development of specific neuronal tissues, such as the mouse retina, long 3′UTRs are also enriched (Hu et al., 2016). The lengthening of the 3′UTRs in neurons is coordinated by the Elav/Hu family of RBPs (Soller and White, 2004). *Drosophila* Elav binds to proximal alternative PASs and thereby promotes the selection of more distal PASs (Hilgers et al., 2012; Carrasco et al., 2020). Interestingly, this process is linked to transcription initiation (Oktaba et al., 2015): Elav binds both to the promoter region, where the RNA polymerase II pauses during transcription initiation, and to the nascent 3'UTR. Both the promoter regions and the RNA polymerase II pausing are necessary for Elav- mediated 3′UTR elongation (Oktaba et al., 2015). Remarkably, in *Drosophila*, upon loss of Elav, a specific splice variant, which is normally repressed by Elav, of the mRNA encoding the RBP “Found in Neurons” (FNE) is produced. The FNE protein encoded by this splice variant can translocate to the nucleus and take over Elav's role in promoting neuronal 3′UTR lengthening (Carrasco et al., 2020).

Why do neurons favor longer 3′UTRs? Since 3′UTRs contain sequences and structural elements that can determine mRNA stability, localization, and translation efficiency, the extension of 3′UTRs increases the number of cis-elements for post-transcriptional regulation of gene expression. Due to the elaborate and complex morphology of neuronal cells and their dendritic and axonal processes, one post-transcriptional mechanism that has prominent functions in neurons is the differential localization and local translation of transcripts, often at very long distances from the cell body. Different 3′UTR isoforms of the same mRNA can thereby localize differentially. In mouse embryonic stem cells and in rat brains, some 3′UTR isoforms are specifically enriched in neuropil regions, i.e., in dendrites and axons, while other isoforms are enriched in the soma or are distributed uniformly (Ciolli et al., 2018; Tushev et al., 2018). Interestingly, the mRNA isoforms with a specific localization usually have a longer 3′UTR, indicating that 3′UTR lengthening confers an enhanced potential for spatial regulation (Tushev et al., 2018). mRNA stabilization and localization are controlled by different RBPs that bind to the 3′UTR, together forming macromolecular complexes called RNA granules. mRNA granules contain several RBPs, such as ZBP1, FMRP, or Staufen2, which are responsible for localization, stabilization, and regulation of translation (Kiebler and Bassell, 2006). mRNA localization and local translation are essential for neuronal development and plasticity (Lin and Holt, 2008; Holt and Schuman, 2013; Shigeoka et al., 2013; Jung et al., 2014; Glock et al., 2017; Cioni et al., 2018a; Biever et al., 2019; Holt et al., 2019), and will be discussed in more detail below.

Alternative cleavage and polyadenylation of specific transcripts controls correct neuronal wiring. Transcripts coding for the murine brain-derived neurotrophic factor (BDNF), which has well-studied roles in axon and dendrite growth and dendrite branching, has either a short or a long 3′UTR (Segal et al., 1995; Cheung et al., 2007; An et al., 2008; Lazo et al., 2013). The neuronal RBP HuD binds specifically to *BDNF* transcripts with the long 3′UTR, and this interaction is necessary and sufficient for selective stabilization of these mRNA molecules, and for elevated expression of BDNF protein (Allen et al., 2013). Moreover, *BDNF* transcripts with short 3′UTRs are restricted to the soma, while the transcripts with long 3′UTR are localized to dendrites. In a mouse mutant with a truncated long 3′UTR, dendritic targeting of *BDNF* mRNAs is impaired (An et al., 2008). This impairment of the long *BDNF* 3′UTR leads to deficits in the pruning of dendritic spines in young mice, suggesting that dendritic targeting of the long *BDNF* isoform controls synaptic connectivity (An et al., 2008).

Interestingly, APA is involved in controlling distinct stages and even opposing processes during axon morphogenesis. Selection of the most distal PAS of the *Drosophila* cell surface receptor Dscam1 is required for axon growth and terminal branching (see below). Conversely, toward the end of axon morphogenesis, selection of the most distal PAS in a component of the cytoskeleton, Ankyrin, mediates stabilization of mature axons and synapses, and growth arrest (Knobel et al., 2001; Pielage et al., 2008). In *Caenorhabditis elegans*, at the end of axon morphogenesis, the casein kinase 1δ (CK1δ) localizes to the nucleus and inhibits transcription termination of *ankyrin*, leading to the production of the longer isoform (LaBella et al., 2020). CK1δ regulates APA by phosphorylating several components of the RNA polymerase-II termination complex (LaBella et al., 2020). Gamma-aminobutyric acid (GABA) motor neurons extend axons from the ventral nerve cord to the dorsal nerve cord during a specific developmental window, after which axon outgrowth stops. Mutations in CK1δ do not affect axon growth, branching, or synaptogenesis during development of GABA motor neurons (LaBella et al., 2020). However, CK1δ mutations lead to continuous elongation of their growth cones after the late larval L1 stage, which leads to a highly branched nervous system (LaBella et al., 2020). The overgrowth phenotype can be suppressed by expression of the giant isoform of Ankyrin, or mutations in the RNA polymerase-II termination complex (LaBella et al., 2020).

**Figure 2:**
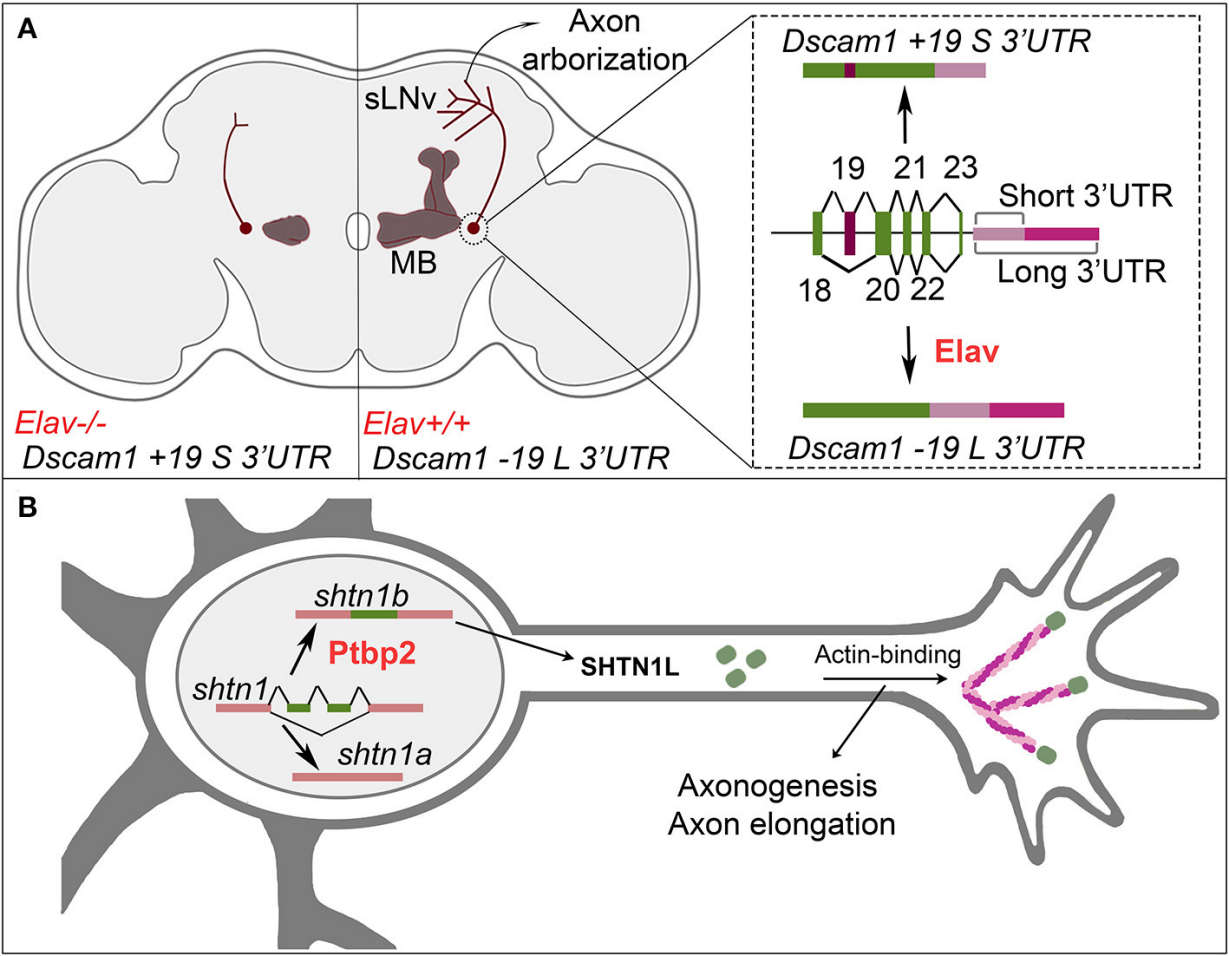
Alternative cleavage/polyadenylation and AS control neuronal wiring. **(A)** Regulation of alternative cleavage/polyadenylation and exon skipping control axon branching and axon arborization. (Right) The RBP Elav mediates the skiping of exon 19 and the selection of the long 3′UTR isoform for *Dscam1*. (Center) The *Dscam1 -19 L 3*′*UTR* is required for bifurcation of *Drosophila* mushroom body (MB) axons, and correct formation of axon arborizations in sLNv neurons. (Left) Loss of Elav or loss of the long 3′UTR of *Dscam1* leads to bifurcation and growth defects in mushroom body axons, and loss of axonal arborizations in sLNv neurons. **(B)** Regulation of AS of *shtn1* coordinates axonogenesis and axon elongation. AS of *shtn1* leads to two different isoforms via the inclusion/exclusion of two exons. The RBP Ptbp2 mediates the inclusion of two exons to produce the *shtn1b/shtn1L* isoform. The SHTN1L isoform binds actin to allow the formation of actin filaments, which leads to axonogenesis and axon elongation. In absence of Ptbp2 or *shtn1L*, axonogenesis is impaired.

3′UTR choice in the mRNA coding for the *Drosophila* Dscam1 cell-surface receptor provides another compelling example of the role of APA in axon morphogenesis. The Dscam1 long 3′UTR is required at a late stage of axon development in the ventral lateral neurons in the fly brain (Zhang et al., 2019b; Figure 2A). Upon specific deletion of the long 3′UTR, the axons of these neurons properly reach their target area, however, they fail to elaborate the typical extensive terminal arborizations in that target area (Zhang et al., 2019b). The RBP Elav binds to the proximal PAS and inhibits its use, thereby promoting the inclusion of the long 3′UTR in the Dscam1 mRNA (Zhang et al., 2019b). Remarkably, the inclusion of the long UTR is coupled by Elav to the exclusion of an alternatively spliced upstream coding exon (Zhang et al., 2019b). It will be exciting to determine in the future whether coupling of APA and AS is a widespread feature during mRNA biogenesis in the developing nervous system.

### 2.2. Alternative Splicing

Alternative splicing is a means to drastically increase the coding potential of genomes. Indeed, many transcripts undergo AS for diversification of the encoded gene products, with the controlled inclusion or exclusion of specific coding exons, or the use of alternative homologous coding exons, ultimately determining the properties of the produced protein isoforms. One of the most remarkable cases of AS in the regulation of neuronal wiring is found for the mRNA encoding the *Drosophila* cell surface receptor Dscam1. Through AS of three alternative exon clusters, more than 18,000 protein isoforms differing in their extracellular domains can be generated from one single gene (12 × 48 × 33 alternative exons for each respective cluster; Schmucker et al., 2000). Dscam1 proteins interact homophilically in a highly isoform-specific manner, with each isoform binding only to itself but not (or very poorly) to other isoforms (Wojtowicz et al., 2004). These interactions mediate neurite self-repulsion, which is required for the assembly of many neuronal circuits. The knowledge on the Dscam1 function has been extensively discussed in several excellent reviews (e.g., Schmucker, 2007; Hattori et al., 2008), and will therefore not be further discussed here. Similarly, in mammals, the Neurexin gene family also encodes a high number of protein isoforms through the use of AS. These presynaptic adhesion molecules instruct synapse formation and the acquisition of cell type-specific and synapse-specific functional properties. A detailed description of Neurexin functions and regulation can be found for example in a recent review (Gomez et al., 2021).

Beyond expanding the coding potential of genes, the AS of both coding exons and of 3′UTRs exons can alter the regulatory elements included in an mRNA, and thus its stability, localization, and translational dynamics. AS events are controlled by RBPs and are often cell-type specific: individual neuron types have highly selective AS programs. These programs were found to control the expression of specific isoforms of proteins that determine intrinsic neuronal excitability, synapse formation and differentiation, pre-synaptic neurotransmitter release, and postsynaptic neurotransmitter sensing (Traunmüller et al., 2016; Furlanis et al., 2019). Through the mRNAs encoding these proteins, AS therefore directly impacts the functional properties of neurons. Remarkably, even closely related neuronal cell types can be reliably distinguished based on their transcript isoform profiles (Furlanis et al., 2019). Importantly, as we will exemplify below, the temporal regulation of AS is an important means to time the expression of specific protein isoforms that will have different effects on neuronal development. The AS programs in most neuronal types are controlled by RBPs from the Nova, Rbfox, Ptbp, Hu/Elav, nSR100, and MbnL families (Raj and Blencowe, 2015; Vuong et al., 2016).

Some RBPs can regulate the splicing of corresponding/paralogous exons in distinct, functionally related genes (Ule et al., 2005; Jacko et al., 2018). Moreover, RBPs direct switch-like changes of the AS program during neuronal development, and the use of specific splicing patterns that are associated either with different stages of neuronal development or of neuronal maturation. For example, in mice, the RBP Ptbp antagonizes more mature splicing programs. This means that for most developmentally regulated exons, it promotes the generation of splicing patterns that are required earlier during development (around 80% of Ptbp-dependent exons). By contrast, Nova, Rbfox, and Mbnl families are generally associated with facilitating splicing programs that are found in more mature neurons (80–96% of target exons of these RBPs; Weyn-Vanhentenryck et al., 2018). The maturation of the AS program is functionally relevant for fundamental aspects of neuronal development. For instance, murine Ptbp2 controls the complex process of axon formation by regulating the AS of axonogenesis-associated genes. In particular, Ptbp2 inhibits the AS switches that stop axon growth, such as for the *shootin1* gene product (Figure 2B). Two isoforms of SHTN1 can be produced by AS. SHTN1L (encoded by the *shootin1b* mRNA isoform) is a protein that binds to both the cell-surface receptor L1-CAM and to actin. By contrast, the isoform SHTN1S (encoded by the *shootin1a* mRNA isoform) does not bind actin (Ergin and Zheng, 2020). SHTN1L promotes actin polymerization in the axonal growth cone and thereby provides a driving force for growth (Toriyama et al., 2006; Zhang et al., 2019a). In early axonogenesis, Ptbp2 inhibits the switch from *shootin1b* to *shootin1a* mRNA isoform usage (Zhang et al., 2019a). The inhibition of this switch maintains axonal growth, and, accordingly, Ptbp2 mutant neurons grow short axons (Zhang et al., 2019a). According to the model, the switch from *shootin1b* to *shootin1a* expression at a later developmental time point (i.e., after axon growth) induces axon specification/maturation mediated by SHTN1S. It is important to note here that the sequence and interdependence of neuritogenesis, axon growth, specification, and maturation remains poorly understood particularly *in vivo*, and that Ptbp2 controls the splicing of other mRNAs coding for proteins involved in these processes. Getting a more complete picture of how switches in alternative splicing programs contribute to axonogenesis represents an exciting challenge for future studies.

Later steps of neuronal development are also controlled by changes in the AS programs. For example, during murine cortical development, Nova2 switches the splicing patterns of mRNAs coding for axon guidance cues and receptors, such as *Dcc, Robo1, Robo2, Slit2*, and *Epha5* (Leggere et al., 2016; Saito et al., 2016; Johnson et al., 2019). Given the essential functions of these cues and receptors, it is not surprising that the loss of *Nova2* leads to severe defects in different parts of the nervous system, such as agenesis of the corpus callosum and impairment of axonal pathfinding of motoneurons and of auditory efferents (Saito et al., 2016). In the murine spinal cord, the two family members Nova 1 and 2 have common RNA targets and function redundantly in regulating the migration of dorsal commissural interneurons, and outgrowth and guidance of their axons toward the ventral midline (Leggere et al., 2016). In this context, Nova1/2 function through *Dcc* splicing (Leggere et al., 2016). Nova1/2 catalyzes the production of a *D*_*cc-long*_ isoform through the choice of an alternative splice acceptor at the 3′ end of a specific intron. Thereby, compared to *D*_*cc-short*_, *D*_*cc-long*_ encodes an additional 20 amino acids in a linker region between two extracellular fibronectin repeats (Leggere et al., 2016). It is not yet fully clear what functional consequences this insertion has. The two murine Dcc isoforms have similar affinities for the Netrin ligand, yet they seem to adopt different conformations upon binding to Netrin (Xu et al., 2014). The D_cc-long_ isoform is clearly implicated downstream of Nova1/2 in spinal cord interneuron axon guidance, as supplying D_cc-long_ suppresses the guidance defects in Nova1/2 double knockout mice (Leggere et al., 2016). Moreover, Nova1/2 provides a typical example of how RNA-binding proteins can be involved in subsequent steps of neuronal development (more precisely, in this case even subsequent steps of axon guidance). After guidance of commissural axons to the ventral part of the spinal cord, they cross the midline to project to the contralateral side of the CNS. The midline represents a typical intermediate target in axon guidance. Intermediate targets need to first attract the axons, before a switch to repulsion happens so that the axons can leave the target and continue their journey. After their function in promoting axon outgrowth and ventral guidance, Nova1/2 promotes midline crossing of spinal cord commissural axons (and thus, axons do not cross the midline in Nova1/2 knockout mice). This is achieved through splicing regulation of a conserved microexon in the transcripts coding for Robo1/2 proteins (Johnson et al., 2019), which are receptors for the classical repulsive cue Slit (Brose et al., 1999; Kidd et al., 1999). Exons that are 3-27 nt long are considered as microexons; in this case, they code for 3 and 4 amino acids in the extracellular domain of Robo1 and Robo2, respectively. Nova1/2 binds to intronic sequences flanking the microexon, and inhibits its inclusion in the *Robo* transcripts. Therefore, in Nova1/2 double knockout animals, only *Robo* transcripts that contain the microexon are expressed. In an elegant genetic experiment, Johnson et al. (2019) deleted the microexon from one allele of each of the *Robo1* and the *Robo2* gene, and thereby restored the expression of both transcript isoforms for each gene in Nova1/2 double knockout mice. Remarkably, this deletion of the microexon in one allele of each *Robo1/2* gene efficiently rescued normal midline crossing, establishing a causal link between Nova1/2-mediated *Robo1/2* splicing and midline crossing. The authors of the study report that the presence or absence of the amino acids encoded by the microexon alter the molecular signaling properties of the Robo receptors. *In vivo*, the Robo1 receptor containing the amino acids encoded by the microexon leads to more axon repulsion than the Robo1 receptor without these amino acids. Consistent with this finding, the microexon splicing is remarkably dynamic in commissural axons, demonstrating the potential of temporal control of AS during neuronal wiring: first, the microexon is included when the axons are guided ventrally, to prevent a premature crossing of the midline. It subsequently gets excluded to allow midline crossing, before it gets included again when the axons have reached the contralateral side, to prevent re-crossing (Johnson et al., 2019).

The third Robo family member, the Robo3 receptor, is also involved in controlling axon midline crossing, and it is also critically regulated by AS (Friocourt and Chédotal, 2017). Robo3 was first identified as generally promoting midline crossing, in contrast to its Robo1/2 paralogs (Sabatier et al., 2004). Later studies however identified a more intricate mechanism. Namely, murine *Robo3* produces 2 isoforms by a rather unusual form of AS: alternative retention of an intron results in different intracellular C-terminal regions between the two encoded receptor isoforms. One of them, Robo 3.1, is expressed in pre-crossing axons of commissural neurons, while the Robo 3.2 isoform is expressed in post-crossing axons (Chen et al., 2008 and see also below). Robo 3.1 is required for midline crossing, while Robo 3.2 contributes to expelling axons from the midline and preventing their recrossing. These data together led to the model that the Robo 3.1 isoform inhibits Robo1/2-mediated repulsion from the midline in pre-crossing axons, while the Robo 3.2 isoform acts in concert with Robo1/2 to mediate repulsion from the midline in post-crossing axons (Chen et al., 2008). Intriguingly, an additional level of complexity is added through a switch that occurred during mammalian evolution, and which eliminated Slit binding to mammalian Robo3 receptors (while Slits bind to Robo3 in non-mammalian vertebrates; Zelina et al., 2014). Instead, mammalian Robo3 interacts with the Netrin-1 receptor DCC and promotes the attraction of commissural neurons to the midline in response to Netrin-1 (Zelina et al., 2014). Therefore, Robo 3.1 could function in pre-crossing axons both by attenuating repulsion and, in mammals, by boosting attraction to midline cues (Blockus and Chédotal, 2016). The precise mechanism of Robo3.2-mediated repulsion in post-crossing mammalian axons and differences in Robo3 functions in distinct types of commissural neurons, remain to be fully addressed.

Alternative splicing also crucially regulates proteins that control synapse formation, specification, and maturation. For example, the “signal transduction and activation of RNA” (STAR) family RBP Sam68 participates in transforming the splicing program of genes involved in synapse development and synaptic transmission in the developing mammalian CNS, including the presynaptic Neurexin cell-surface receptors and several postsynaptic scaffolding proteins (Iijima et al., 2011; Witte et al., 2019; Farini et al., 2020). Sam68 mainly functions by preventing exon inclusion. In the cerebellum of mice lacking Sam68, there is increased inclusion of exons, and this impairs the maturation of cerebellar Purkinje cells and leads to a reduction of synaptic contacts between Purkinje cells and granule cells (Farini et al., 2020). The mammalian cerebellum critically contributes to motor and social behaviors. Therefore, it is not surprising that the connectivity defects in cerebellar circuits lead to dysfunction in these behaviors (Farini et al., 2020), and that they could contribute to the association between alternatively spliced Sam68 targets and autism-spectrum disorders.

Liquid-liquid phase separation has recently emerged as a cellular mechanism implicated in synapse formation, function, and plasticity (Milovanovic et al., 2018; McDonald et al., 2020; Hosokawa et al., 2021). AS can contribute to the regulation of liquid-liquid phase separation, as exemplified by the SynGAP protein, an abundant component of the postsynaptic density (Zeng et al., 2016). SynGAP negatively regulates synaptic strength, and downregulation of SynGAP leads to premature formation of enlarged spines in the hippocampus of young mice (Vazquez et al., 2004; Clement et al., 2012). Moreover, some forms of long-term synaptic potentiation lead to SynGAP dispersion from the postsynaptic density. Rat SynGAP binds to another postsynaptic density protein, PSD95. This interaction induces phase separation of the SynGAP/PSD95 complex, and it is required for maintaining SynGAP localization in the postsynaptic density (Zeng et al., 2016). Complex AS of murine *SynGAP* pre-mRNA generates protein isoforms with differences in their C-terminal domain (McMahon et al., 2012). Of these, only the isoform α1 was able to induce liquid-liquid phase separation in a heterologous cellular assay, while the α2, β, and γ isoforms were not (Araki et al., 2020). Consistent with these biochemical properties, synaptically localized murine SynGAP α1 was rapidly dispersed upon LTP induction, while SynGAP β was less enriched at synapses and did not disperse during LTP. SynGAP α1 dispersion from the synapse is required to allow dendritic spine enlargement and insertion of AMPA-type glutamate receptors during LTP, suggesting that SynGAP α1 is the main isoform involved in this type of synaptic plasticity, while the other isoforms play at best modest roles in this process. By contrast, the “division of labor” between isoforms is different in another major neuronal SynGAP function, namely the control of developmental dendrite morphogenesis and maturation (Aceti et al., 2015; Araki et al., 2020). Indeed, only the SynGAP β isoform supports the normal branching of distal dendrites (Araki et al., 2020). Remarkably, disrupting the propensity of SynGAP α1 to undergo liquid-liquid phase separation rendered this isoform capable of taking over the function of SynGAP β in controlling distal dendrite morphogenesis. These results directly link the different phase separation characteristics to separable neuronal functions of SynGAP isoforms, and exemplify how AS can generate protein isoforms with distinct biochemical properties that underlie different cellular functions.

Moreover, the case of the SynGAPs provides an additional example of how AS can contribute to the regulation of protein biogenesis beyond generating mRNAs with different coding sequences and thus proteins with different properties: *syngap* mRNA isoforms are differentially stabilized post-transcriptionally, which contributes to regulating relative expression levels of the different isoforms, potentially underlying differences in expression of the SynGAP isoforms at distinct developmental stages. More specifically, the 3′UTR of the murine *syngap* α*2* mRNA includes binding elements for the RBP FUS, which are not present in the *syngap* α*1* mRNA (Yokoi et al., 2017). The binding of FUS and also ELAV4 to the *syngap* α*2* 3′UTR leads to stabilization of *syngap* α*2* mRNA and higher SynGAP α2 protein levels (Yokoi et al., 2017). In the absence of ELAV4 binding, FUS dissociates from *syngap* α*2* mRNA (Yokoi et al., 2017). In this situation, the ELAV1 family member binds to the *syngap* α*2* mRNA, which correlates with a decrease in SynGAP α2 protein levels (Yokoi et al., 2017). Interestingly, murine FUS also regulates the stability of the *GluA1* mRNA. FUS binds to the 3′UTR of *GluA1* and enhances polyadenylation of the mRNA, which correlates with higher GluA1 protein levels (Udagawa et al., 2015). Functionally, the absence of FUS leads to impaired maturation of dendritic spines, and this phenotype can be rescued by the expression of SynGAP α2 or GluA1 (Udagawa et al., 2015; Yokoi et al., 2017). Thus, a common RBP regulates the metabolism of different mRNA targets whose products are involved in a common neurodevelopmental process.

### 2.3. RNA Localization and Local Translation

Local translation has long been recognized to account for site-specific protein production in dendrites and at post-synapses. *In vivo* evidence for local translation in (developing) axons has however emerged more recently, but has been the subject of several excellent reviews. We will thus not discuss it extensively here. In axonal compartments, local translation allows for a localized and fast remodeling of the axonal proteome (Lin and Holt, 2008; Holt and Schuman, 2013; Shigeoka et al., 2013; Jung et al., 2014; Glock et al., 2017; Cioni et al., 2018a; Biever et al., 2019; Holt et al., 2019). Local translation occurs at many steps of axonal wiring. In the early steps, during axon growth and targeting, specific guidance cues rapidly up- or down-regulate a large number of locally translated proteins. Repulsive and attractive cues can thereby generate opposite remodeling of axonal proteins (Cagnetta et al., 2018).

A prerequisite for local translation is, obviously, the localization of mRNAs to specific subcellular compartments. mRNA localization and local translation have a large impact on the proteome in neuronal processes. Indeed, nearly half of the proteins in the neurite-enriched proteome are locally translated (Zappulo et al., 2017). Thereby, axonal mRNAs that encode key regulators of axonal outgrowth, branching and synaptogenesis are dynamically localized and translated during CNS development (Shigeoka et al., 2016). This mRNA localization is developmentally regulated: growing axons contain a different set of mRNAs than mature axons (Gumy et al., 2011). In neurons, the distribution of mRNA is differentially regulated not only between dendrites, cell body, and axons, but also to a finer spatial level within axons: sub-axonal compartments, such as the axon shaft, the central domain of the growth cone, and the peripheral domain of the growth cone, respectively, contain different subsets of localized mRNAs (Zivraj et al., 2010; Wang et al., 2014). Active transport contributes to the differential localization of mRNAs in sub-axonal compartments (Turner-Bridger et al., 2018). In this section, we will first introduce general mechanisms for mRNA localization, highlighting some examples that have emerged recently. We will then discuss instances of mRNA localization and local translation in developing neurons.

mRNAs are transported in ribonucleoprotein (RNP) complexes that typically contain a couple dozen RBPs, such as helicases and regulators of translation, and also non-coding RNAs with regulatory functions (Fritzsche et al., 2013; Mitchell and Parker, 2014). RNP complexes can be further assembled into bigger structures for transport, known as RNP granules (Mitchell and Parker, 2014). The formation of RNP complexes is mediated by protein-protein interactions leading to oligomerization, or liquid-liquid phase separation driven by intrinsically disordered protein domains (IDDs; also known as low complexity regions). RBPs typically contain IDDs (Weber and Brangwynne, 2012). In the context of RNPs, IDDs have functions beyond assembly: for example, the IDD of *Drosophila* IMP (a homolog of the vertebrate ZBP1) is not required for RNP assembly, but rather for the regulation of the dynamics and other properties of RNPs (Vijayakumar et al., 2019). The Imp IDD modulates the size, the number and the motility of RNP granules, and in the *Drosophila* CNS, it regulates the transport of RNP granules to axons during development. This is a key mechanism for the proper remodeling of the axons of mushroom body γ neurons (Vijayakumar et al., 2019).

The localization of mRNAs to neurites can be achieved through different mechanisms of transport. One of them is the directional transport to axons and dendrites *via* RNP anchoring to motor proteins (Abouward and Schiavo, 2021). Motor proteins, such as the Dynein and Kinesin families, move along the microtubule cytoskeleton, and can deliver RNA cargo to distal neuronal processes (Kanai et al., 2004). The minimal array of elements described to be sufficient for proper mRNA localization to axons consists of a kinesin motor protein (Kinesin-2), an adaptor protein (KAP3), and an RBP (adenomatous polyposis coli, APC; Baumann et al., 2020). *In vitro*, these components are sufficient for delivering β-actin and β2B-tubulin mRNAs to the axonal terminal. Baumann et al. (2020) further identified that one to three mRNA molecules are found in a single RNP transport complex. Moreover, it suggests that other proteins, which are present in a single RNP, may have functions that are not directly related to transport.

Recently, two more mechanisms of transport were described in neurons. First, in mammalian cells, RNAs can be transported by hitchhiking onto motile late endosomal/lysosomal organelles (Liao et al., 2019). Using a proximity ligation assay, Liao et al. (2019) identified that one of the proteins mediating the anchoring of RNP complexes to late endosomes is annexin A11 (ANXA11). ANXA11 contains an IDD in its N-terminal domain that mediates liquid-liquid phase separation and formation of the RNP complexes. In the C-terminal part of ANXA11, a membrane binding domain tethers the protein to the membrane of endosomes (Liao et al., 2019). Tethering of RNPs to endosomes for transport and local translation in *X. laevis* retinal ganglion cell axons suggest that this mechanism is shared among vertebrates (Cioni et al., 2019). The second unconventional mechanism of transport is mediated by extracellular vesicles. Here, proteins, RNA, and other molecules are encapsulated in secreted vesicles and transported between different cells and cell types in the nervous system (Morel et al., 2013; Xu et al., 2017; Ashley et al., 2018; Pastuzyn et al., 2018). For instance, mammalian and *Drosophila* Arc have properties resembling retroviral Gag proteins: Arc proteins form structures similar to virus capsids, which are used to encapsulate mRNA. The *Arc* mRNA is deposited inside an Arc capsid, and the capsid is transported in extracellular vesicles across synaptic partners (Ashley et al., 2018; Pastuzyn et al., 2018). This transport mechanism is required for developmental and activity-dependent synapse morphogenesis at the *Drosophila* NMJ (Ashley et al., 2018). However, how intercellular RNA transfer contributes to the axonal and dendritic proteome, as well as its impact on neuronal wiring and synaptogenesis, remains to be fully addressed.

All three above-mentioned mechanisms of transport involve at least one RBP that selects mRNA targets based on specific binding elements in the RNA sequence. The incorporation of these elements is developmentally and spatially regulated by different mechanisms, such as AS. For example, this is the case of the Staufen (Stau)-mediated transport of *calmodulin3* (*calm3*) and *CaMKII* α mRNAs in the mammalian brain (Ortiz et al., 2017, Sharangdhar et al., 2017). The mRNA of *calm3* localizes to dendrites upon binding of Stau2 to an intron retained in the *calm3* isoform with the longest 3′UTR (Sharangdhar et al., 2017). Similarly, the mRNA of the *CaMKII* α isoform that retains intron 16 is bound by Stau2 and subsequently localized to dendrites (Ortiz et al., 2017). Importantly, active mechanisms are also used to avoid erroneous transport to neuronal processes. For instance, in the mammalian brain, mRNAs containing Pumilio2 (Pum2) binding elements are retained in the cell body, and ectopic translation of these mRNAs is avoided during the early stages of development. Later, at the adult stage, when the expression of Pum2 decreases, these mRNAs become enriched in the axonal compartment and locally translated (Martinez et al., 2019). Thus, the regulation of RNA localization is a key feature of CNS development. In the next section, we will illustrate how several steps of neuronal wiring, namely axon growth, axon branching, and synaptogenesis, are controlled by differential localization and translation of mRNAs.

Directional switches during axon guidance are based on axonal growth cone turning toward attractive cues and away from repulsive cues. These responses are mediated by the stabilization of cytoskeletal elements in the growth cone compartment that is exposed to attractive cues, and, conversely, to the destabilization of the cytoskeleton in the compartment that is exposed to repulsive cues (Terenzio et al., 2017). During axon guidance, β*-actin* mRNA undergoes local translation *in vivo* in axons of *X. laevis* (Wong et al., 2017). The zip-code binding protein 1 (ZBP1) homolog Vg1RBP binds to the 3′UTR of β*-actin*, and transports the β*-actin* mRNA to growth cones and within growth cones (Leung et al., 2006). Vg1RBP and β*-actin* transcripts move into filopodial protrusions of growth cones upon Netrin-1 induced attraction. Moreover, Netrin-1 or BDNF can induce asymmetrical β-actin translation in the growth cone, which leads to directional turning of growth cones during axon guidance (Leung et al., 2006; Yao et al., 2006; Welshhans and Bassell, 2011). Mediators of actin disassembly are also regulated by cue-induced local translation. For instance, in *X. laevis*, transcripts encoding the actin filament-severing protein Cofilin are locally translated upon exposure to the repulsive cue Slit-2, inducing growth cone collapse (Piper et al., 2006). Moreover, in chicken, upon semaphorin-3A (Sema3A) exposure, the GTPase RhoA and the RhoA-kinase on the one hand inhibit actin polymerization-dependent formation of protrusions, thereby enhancing growth cone collapse. On the other hand, chicken RhoA-kinase promotes the formation of intra-axonal F-actin bundles that mediate myosin II-dependent retraction (Dontchev and Letourneau, 2002; Wu et al., 2005; Gallo, 2006). In the rat, the activation of this pathway occurs through local axonal translation: transcripts of *RhoA* localize to developing growth cones, and Sema3A induces its local translation, and thus growth cone collapse (Wu et al., 2005).

Local translation is also key for axon branching. In *X. laevis* retinal ganglion cells, Vg1RBP/ZBP1 localizes to regions of filopodia sprouting and it is required for the formation of terminal arborizations (while it is not required for long–range axon navigation; Kalous et al., 2014). Likewise, β*-actin* mRNA is transported in RNA granules that dock at sites of new branch emergence, and its local translation is required for terminal axon branching (Wong et al., 2017). Moreover, in chicken embryonic sensory axons, the formation of new branches is supported by the local translation of the actin-nucleation complexes Arp2, WAVE1, and cortactin, which are essential for both the formation of actin patches and for filopodia emergence from them (Spillane et al., 2012). Interestingly, another regulator of actin assembly, Mena (also called ENAH; Krause et al., 2003), interacts with several RBPs and mRNAs in murine axonal growth cones, forming ribonucleoprotein complexes that also include *Mena* mRNA itself (Vidaki et al., 2017). Mena is required for local translation of the mRNAs present in those complexes (Vidaki et al., 2017). Therefore, Mena regulates both, actin dynamics and local translation, linking the two processes. Besides mRNAs coding for regulators of actin remodeling, other mRNAs, mitochondria, and ribosomes are located to axon branch points. The local translation of mitochondrial and ribosomal proteins, as well as ribosome assembly and mitochondria function in axons, support branch formation (Courchet et al., 2013; Spillane et al., 2013; Wong et al., 2017; Cioni et al., 2019; Shigeoka et al., 2019).

Recent studies showed how key components of the synapse are locally translated during synapse assembly. For example, rat *SNAP25* (encoding a component of the SNARE complex that is involved in the release of synatic vesicles) and β*-catenin* (encoding a subunit of the Cadherin/β-Catenin complex that is involved in cell adhesion) are locally translated during the formation of presynapses, and their protein products cluster with presynaptic proteins (Taylor et al., 2013; Batista et al., 2017). However, relatively little is known about the functions of local translation in other aspects of synapse formation, such as the subcellular control of synaptogenesis, synapse specification, and synaptic partner choice. Intriguingly, a recent study on the *Drosophila* membrane-associated dual-specificity “phosphatase of regenerating liver-1” (Prl-1) suggests that local translation may be involved in controlling axon compartment-specific synaptic connectivity (Urwyler et al., 2019). Prl-1 promotes high local synapse number in one specific collateral branch of a *Drosophila* CNS axon. Both, this function and Prl-1 protein enrichment in this axon collateral branch, depend on the UTRs of the *prl-1* mRNA. UTR-dependent localization of the Prl-1 protein to a specific axon compartment thus suggests that local translation may be a key mechanism to confer spatial specificity of Prl-1 function to this compartment. Remarkably, for promoting high local synapse number, *prl-1* genetically interacts with components of the InR/Akt signaling pathway and its downstream effector, the mTOR complex (Urwyler et al., 2019). One major output of the InR/Akt/mTOR axis is the control of translation (Roux and Topisirovic, 2018), suggesting that Prl-1 may contribute to the regulation of local translation of both its own mRNA (in a positive feedback loop) and of other mRNAs localized to that axon compartment. This is reminiscent of mTOR-dependent local translation of *mTOR* mRNA, and other mRNAs, in injured axons (Terenzio et al., 2018). Further studies are required to test this model of compartmentalization of Prl-1 localization and function through local translation. Moreover, it will be exciting in the future to decipher additional mechanisms that depend on local translation for controlling axon compartment-specific synaptogenesis, synapse specification, and synaptic partner matching in the CNS.

### 2.4. RNA Modification, Non-Coding RNAs, and Decay Mechanisms in the Control of Local Axonal Translation

**Figure 3:**
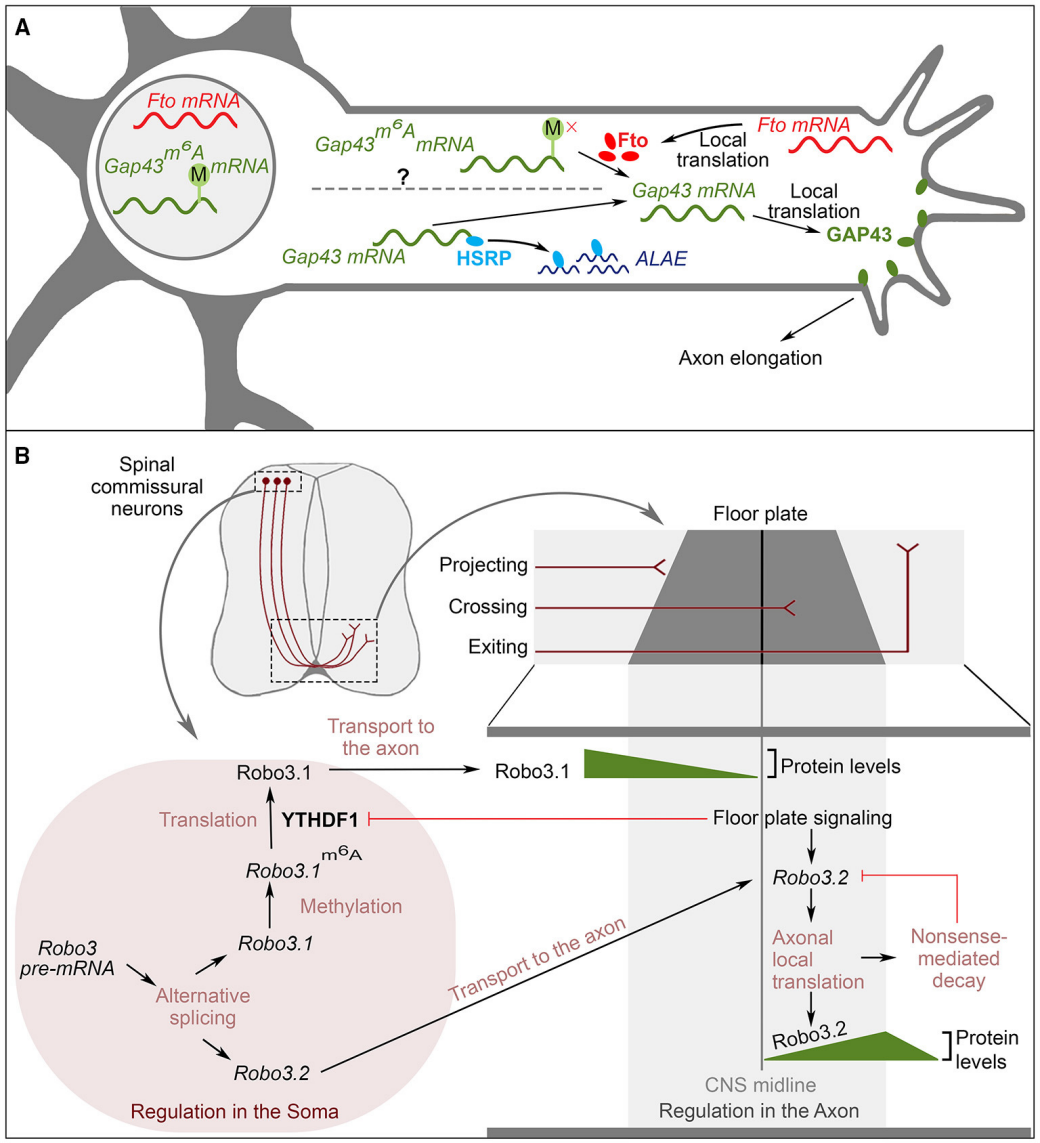
The interplay of RNA modification and local translation for neuronal wiring **(A)** Regulation of local translation of *Gap43* mRNA for axon elongation. *FTO* mRNA is locally translated in axons. The eraser FTO mediates the de-methylation of *Gap43*^*m*^*6*^*A*^ *mRNA* in axons. This leads to local translation of *Gap43* mRNA. *Via* an alternative pathway, HSRP binds to the 3′UTR of *Gap43* mRNA to repress its translation. The non-coding RNA ALAE is enriched in the axon and sequesters HSRP. Released *Gap43* mRNA can then be translated locally. *Gap43* associates with the plasma membrane and regulates actin dynamics to promote axon elongation. The question mark on the dotted line highlights that it is unknown whether these two pathways interact. **(B)** RNA metabolism of Robo3 involved in midline crossing of commissural neurons. (Top) Cross-section of spinal cord showing the trajectory of commissural neurons. During development, these neurons project their axons ventrally, where they cross the midline to target the contralateral side. (Bottom) *Robo3* RNA metabolism leads to spatially and temporally controlled expression of Robo3 during navigation of commissural axons. (Left) Events occurring in the soma. (Right) events occurring in the axons. *Robo3* can produce 2 isoforms *via* AS, *Robo3.1* and *Robo3.2*. From these isoforms, *Robo3.1* is methylated, and the m^6^A modification is read by YTHDF1, which stimulates translation of *Robo3.1*^m^6^A^ in the soma. When commissural axons reach the floor plate, signaling from the floor plate downregulates YTHDF1 expression, thus inhibiting *Robo3.1* expression in post-crossing axons. At this point, floor plate signaling induces the local translation of the *Robo3.2* isoform. After translation, *Robo3.2* is degraded by nonsense-mediated decay. Growth cones projecting to the floor plate express Robo3.1 only. After reaching the floor plate, the protein levels of Robo3.1 decrease and Robo3.2 protein levels increase. Shortly after crossing the midline, Robo3.2 protein levels are downregulated.

Internal chemical modification of mRNAs, often referred to as “epitranscriptomics,” is a major way to control gene expression (Frye et al., 2018). Of the more than 170 different known RNA modifications, *N*^6^-methyladenosine (m^6^A) has attracted particular attention as a major regulator of translation. m^6^A is a reversible and dynamic modification that is controlled by methyltransferases (“writers,” which add the modification) and demethylases (“erasers,” which remove the modification; Roundtree et al., 2017). Within specific sequence contexts, the modification is recognized by RBPs called “readers” (Roundtree et al., 2017). “Writing and erasing” of m^6^A in mRNA is involved in controlling local translation in axons (Yu et al., 2017). For example, in rat dorsal root ganglion (DRG) neurons, the local translation of *Gap-43* mRNA, which is required for axon elongation, is regulated by *N*^6^A methylation (Donnelly et al., 2013). Intracellular, cell membrane-associated GAP-43 protein induces growth *via* regulation of actin dynamics (Laux et al., 2000; Denny, 2006). The murine *Gap-43* mRNA is *N*^6^A-methylated in the cell body and then transported to the axon in a translationally repressed state (Figure 3A; Yu et al., 2017). An m^6^A eraser, FTO, is locally translated in axons, and locally removes the *N*^6^A methylation from *Gap-43* mRNA, which derepresses its translation (Figure 3A; Yu et al., 2017). Upon loss of FTO, m^6^A modifications in *Gap-43* mRNA remains present, and *Gap-43*^*m*^*6*^*A*^ mRNA accumulates (Yu et al., 2017). This modified mRNA is not translated locally, which results in reduced GAP-43 protein levels in the axon and failure of axon elongation (Yu et al., 2017). Interestingly, there is a second, independent mechanism regulating the local translation of *Gap-43* mRNA. ALAE, an axon-enriched long intergenic non-coding RNA, also controls *Gap-43* local translation, as observed in rat DRG axons (Figure 3A; Wei et al., 2021). More specifically, under normal conditions, ALAE binds to an RBP called KHSRP in the axonal compartment. KHSRP that is not bound to ALAE can bind to the 3′UTR of *Gap-43* mRNA and inhibit *Gap-43* translation without affecting its mRNA levels (Wei et al., 2021). ALAE functions as an “RNA-decoy” for KHSRP because ALAE-bound KHSRP cannot bind to the 3′UTR of *Gap-43* mRNA. Consistent with this model, in the absence of ALAE, protein levels of rat GAP-43 are reduced and axon elongation is impaired. These phenotypes are recapitulated by a disruption of the ALAE-KHSRP interaction, which does not affect RNA levels of either ALAE or *Gap-43* (Wei et al., 2021). Therefore, ALAE promotes axon elongation by preventing KHSRP-mediated inhibition of *Gap-43* mRNA translation (Wei et al., 2021). Together, these mechanisms exemplify the dynamic control of local translation in the axon, which in turn is crucial for local regulation of the axon cytoskeleton during neuronal wiring. In the cell body and possibly during the transport of *Gap-43* mRNA, translation is inhibited both by *N*^6^A methylation and by KHSRP binding. Once the *Gap-43* mRNA has reached the axon, m^6^A- and KHSRP-mediated inhibition of translation are both removed by FTO and ALAE action, respectively. More studies are required to investigate whether these two mechanisms interact, and how ALAE is localized to the axon. Both m^6^A modification and KHSRP binding occur in the 3′UTR of *Gap-43* mRNA (Yu et al., 2017; Wei et al., 2021), highlighting again the pivotal role of 3′UTRs as regulatory hubs in neuronal wiring.

Methylation of *Robo3* mRNA further exemplifies the role of RNA modification in neuronal wiring. In mice, the guidance of commissural neuron axons across the midline is regulated by the Robo 3 receptor, for which two isoforms are produced by AS (Chen et al., 2008). As described above, Robo 3.1 localizes to the pre-crossing axonal segment of commissural neurons, while the Robo 3.2 isoform localizes to the post-crossing axonal segment (Chen et al., 2008). The spatial regulation of Robo 3.1 and 3.2 is achieved by local translation (Figure 3B; Colak et al., 2013). Both mRNA isoforms are present in the growth cone. Before midline crossing, Robo 3.2 translation is repressed, and only Robo 3.1 is expressed (Colak et al., 2013). Expression of the Robo 3.1 protein needs continuous local translation, because of the short half-life of the protein (Zhuang et al., 2019). To increase Robo 3.1 protein levels in pre-crossing axons, *Robo 3.1* mRNA is *N*^*6*^*A*-methylated and bound by the m^6^A reader YTH domain-containing family 1 (YTHDF1), which positively regulates *Robo 3.1* mRNA translation (Zhuang et al., 2019; note that in this case, m^6^A is stimulating translation, while in the case of GAP-43 described above, m^6^A is inhibitory). When axons reach and cross the midline, floor plate signaling down-regulates YTHDF1 and thereby reduces *Robo 3.1* expression (Zhuang et al., 2019). As a result, Robo 3.1 levels are higher in the pre-crossing axon segment than in the post-crossing segment. At the same time, floor plate signaling induces local translation of Robo 3.2 transcripts (Colak et al., 2013). After the first round of translation, however, mRNAs are targeted for nonsense-mediated decay (NMD), which gradually reduces Robo 3.2 protein levels in post-crossing axons (Colak et al., 2013). These sophisticated regulatory mechanisms ensure that *Robo 3.1/3.2* expression is spatially tightly restricted: only *Robo 3.1* is expressed before crossing the midline, and *Robo 3.2* is expressed only in a short post-crossing axonal segment. Robo 3.1 attracts the axons to the midline, and allows midline crossing (Zelina et al., 2014). Robo 3.2 was suggested to mediate repulsion from the midline (Colak et al., 2013). Its localization only in the post-crossing segment avoids early repulsion (Colak et al., 2013). The expression of Robo 3.2 induced by the floor plate allows axons to exit the midline (Colak et al., 2013). In turn, the continuous decay of Robo 3.2 in the post-crossing axonal segment prevents over-repulsion from the midline, and sets the distance from the midline, at which the axons will turn rostrally and continue their journey in the contralateral side of the CNS (Chen et al., 2008; Colak et al., 2013; Zhuang et al., 2019).

Another molecular mechanism involving m^6^A reading was identified in *Drosophila*, where the YTHDF1 homolog (YTHDF) interacts with the RBP FMRP. Murine FMRP binds polyribosome-associated mRNAs and can inhibit translation by promoting ribosome stalling (Darnell et al., 2011). *Via* this, and other mechanisms, FMRP is a negative regulator of local translation with large impacts on neuronal wiring (Davis and Broadie, 2017). FMRP is expressed in almost all neuronal cell types and localizes to axons and to pre-synapses during synaptogenesis (Christie et al., 2009). At the *Drosophila* neuromuscular junction (NMJ), loss of FMRP leads to increased axonal growth due to increased translation of the *chic* mRNA (Zhang et al., 2001; Reeve et al., 2005). *chic* encodes Profilin, an actin-binding protein that promotes axonal outgrowth (Wills et al., 1999). A recent study identified several shared mRNA targets of YTHDF and FMRP, including *chic* and *futsch* (the latter encoding a microtubule-associated protein that regulates axon growth at the NMJ; Worpenberg et al., 2021). YTHDF stabilizes FMRP binding to these mRNAs, which represses their translation. This interaction thereby limits axon growth both at the NMJ and in the CNS (Worpenberg et al., 2021). A major open question to tackle in the future is whether YTHDF homologs in mammals can also repress translation depending on the involved target and interaction partners, and, conversely, whether *Drosophila* YTHDF can also stimulate translation (Worpenberg et al., 2021).

### 2.5. RNA Editing

The most frequent form of RNA editing is adenosine deamination (Adenosine-to-Inosine, A-to-I editing), catalyzed by the ADAR family of RBPs. The produced inosines are read as guanosines by cellular proteins, and this can alter codons and splicing events, thus leading to changes in protein function (Tariq and Jantsch, 2012; Nishikura, 2016; Walkley and Li, 2017). A to I editing is most abundant in the CNS (Ramaswami et al., 2013) and increases progressively during development (Hwang et al., 2016). Like other regulatory mechanisms of RNA metabolism, RNA editing is cell type-specific (Lundin et al., 2020). ADARs are enriched in the nucleus at different stages of brain development (Desterro et al., 2003; Behm et al., 2017). This led to the hypothesis that mRNA editing is restricted to the nucleus. However, RNA editing can also occur in the cytoplasm, such as in adult axons of the squid (Vallecillo-Viejo et al., 2020). Surprisingly, in these neurons, the rate of editing is higher in axons than in cell bodies (Vallecillo-Viejo et al., 2020). The presence and function of RNA editing in developing axons, such as at the stage of axon outgrowth and targeting, are still to be discovered.

Among the neuronal proteins recoded by editing, neurotransmitter receptors, ion channels and other genes involved in rapid electrical and chemical transmission are the principal identified targets of ADARs (Hoopengardner et al., 2003). The rat 2C subtype of serotonin receptors (5-HT2CR) is edited in the intracellular domain, which leads to a dramatic decrease in signaling downstream of the receptor (Burns et al., 1997). In mice, the α3 subunit (*Gabra-3*) of the GABA_A_ receptor is edited by ADARs, replacing isoleucine with methionine in the transmembrane region of the protein (Ohlson et al., 2007). This editing event depends on an intronic stem loop 150 nt downstream of the edited site (Daniel et al., 2012). The amino acid change decreases α3 protein levels and trafficking to the cell membrane (Daniel et al., 2011). The reduction of cell surface presentation of the edited α3 subunit is mediated by both, higher receptor internalization from the membrane and degradation by the lysosomal pathway (Daniel et al., 2011). The recoding also affects the biophysical properties of the channel: editing leads to higher sensitivity to GABA and faster deactivation (Nimmich et al., 2009). The *Drosophila* homolog of the GABA receptor is formed by homomers of RDL (resistance to dieldrin), whose encoding transcript is diversified into 4 alternative variants due to AS (Ffrench-Constant and Rocheleau, 1993). Additional isoform diversity is given by RNA editing that changes four amino acid residues (Hoopengardner et al., 2003; Jones et al., 2009). The recoding and the choice of alternative exons are linked, and depend on the developmental stage, although the underlying molecular mechanisms are unknown (Jones et al., 2009). As in the mammalian receptor, the combination of AS and amino acid recoding determine the functional properties of the receptor in *Drosophila* (Jones et al., 2009). In mammals, the glutamate receptors GluA1, GluA2, GluA3, GluA4, GluA5, and GluA6 (encoded by *Gria1, Gria2, Gria3, Gria4, Gria5, and Gria6*, respectively) are also edited by ADARs (Bernard and Khrestchatisky, 1994; Bass, 2003). The RNA editing rates of the transcripts of these receptors change from early development until adulthood, leading to the expression of distinct receptors that differ in single amino acids across development (Wahlstedt et al., 2009). Similarly to GABA receptors, recoding of glutamate receptors also leads to changes in functional properties, namely lower permeability (Egebjerg and Heinemann, 1993), faster recovery after desensitization (Lomeli et al., 1994), and also a decrease in the insertion rate into the plasma membrane (Araki et al., 2010).

Besides their function in synaptic transmission, neurotransmitter receptors also have functions in synaptogenesis and neuronal wiring. In chicken, the GluA2 subunit of AMPA receptors is required for the formation of dendritic arborizations (Yoon et al., 2012), and in mammalian brains, it can modulate spine formation (Saglietti et al., 2007; Lee et al., 2016). The murine GABA_A_ receptor can induce synaptogenesis (Oh et al., 2016), and it is also required for spine formation (Heinen et al., 2003). Since RNA editing modifies the properties and membrane insertion of GluA2 and GABA_A_, RNA editing could potentially affect neuronal wiring *via* these neurotransmitter receptors. Whether and how this is the case remains a major unsolved question that should be addressed in future studies.

Adenosine deaminases that act on RNA also have other targets with more evident functions in neuronal wiring. Among them are *Filamin-*α (FLNa), *Filamin-*β (FLNb), and *Nova1* (Tariq and Jantsch, 2012; Nishikura, 2016). FLNa and FLNb are actin binding proteins that control actin reorganization and are required for neurogenesis, neuronal migration, and axon guidance (Fox et al., 1998; Zheng et al., 2011; Zhang et al., 2013; Oliva et al., 2015). *Drosophila* FLNa and FLNb interact with the cell surface receptors Teneurin-2 (Ten-m) and Semaphorin-1a (Sema-1a) and mediate responses downstream of receptor activity (Zheng et al., 2011; Jeong et al., 2017; DePew et al., 2019). *Via* interactions with FLN, Ten-m controls growth cone guidance (Zheng et al., 2011; DePew et al., 2019). Moreover, a bioactive peptide corresponding to the C-terminal region of the protein is produced from the Ten-m locus, and it strongly induces filopodia formation and growth cone enlargement through interaction with FLN (Rubin et al., 1999). Sema-1a promotes axon outgrowth and, depending on the context, is an attractive axon guidance cue (such as in grasshoppers; Wong et al., 1997, 1999), or a repulsive axon guidance cue through interaction with FLN (such as in *Drosophila*; Jeong et al., 2017). However, it is still unknown if editing of *FLN* mRNA affects its response to receptor activity. Nova1 regulates AS of several receptors required for correct neuronal wiring (see section above), and although the editing of murine *Nova1* had no direct impact on the splicing activity in a heterologous system, it leads to reduced proteasome targeting and extended half-life of Nova1 (Irimia et al., 2012). How Nova1 editing is regulated to control neuronal wiring remains elusive.

Although it is not yet known if its editing affects its function in neuronal wiring, the mRNA encoding the cytoplasmic FMRP-interacting protein 2 (CYFIP2) constitutes an intriguing target of ADARs in the contexts of circuit development (Tariq and Jantsch, 2012; Nishikura, 2016). CYFIP2 is a member of the WAVE complex that can trigger actin nucleation, and it is required for axon guidance and synaptogenesis (Schenck et al., 2003; Zhao et al., 2013). Interestingly, CYFIP2 interacts with the RBP FMRP (see above) in the growth cone and mediates actin remodeling, for example in the context of optic tract axon sorting (Schenck et al., 2001, 2003, 2004; Cioni et al., 2018b). Remarkably, in both vertebrates and invertebrates, FMRP interacts directly with ADAR and regulates ADAR RNA editing activity, particularly of synaptic proteins (Shamay-Ramot et al., 2015; Filippini et al., 2017). In zebrafish, ADAR and FMRP interact biochemically, and FMRP limits axon branching and synapse density in different projection, sensory and motor neurons (Shamay-Ramot et al., 2015). At the *Drosophila* NMJ, the knockout of either *Fmr1* or *Adar* leads to an increase both in axon branching and in synaptic boutons, and a reduction of postsynaptic GluRIIA receptor levels (Bhogal et al., 2011; Maldonado et al., 2013). Yet, FMRP is not the only RBP that modulates ADAR activity: a recent *in vivo* genetic screen in *Drosophila* identified several such RBPs, including Rbp6, a Musashi family protein (see below), and Pasilla, a Nova1/2-homolog best described as a splicing regulator (Sapiro et al., 2020). The evolutionarily conserved zinc finger protein Zn72D, however, turned out as the major regulator of ADAR-mediated RNA editing, affecting almost 60% of the investigated editing sites, mostly stimulating their editing. Consistently, knockout of Zn72D leads to a similar reduction of GluRIIA receptor levels at the *Drosophila* NMJ as ADAR knockout (Sapiro et al., 2020). In total, this study identified more than 1,200 editing sites in introns, untranslated regions, and coding sequences, with editing efficiencies (i.e., the fraction of mRNA molecules with an edited nucleotide) ranging from a few percent to a hundred percent. A major challenge for the field of RNA editing is the investigation of the effects on protein expression and function, and neuronal wiring, of each of these editing sites.

## 3. The Musashi RNA Binding Protein Family as Master Regulators of Neuronal Development

A intriguing and debated question in the field of neuronal wiring is how the differential regulation of gene expression can generate the highly specialized protein repertoires, which are needed for correct circuit formation, in a cell type-specific and temporally controlled manner. Individual RBPs typically act on hundreds of RNA targets to post-transcriptionally regulate different aspects of mRNA metabolism, and, concomitantly, different stages of neuronal wiring. The evolutionary conserved Musashi (Msi) protein family constitutes a prime example of this diversity in RBP molecular function and repeated involvement in neuronal development. Msi proteins control neural stem cell maintenance, neuronal proliferation and differentiation, neuronal morphology, axon guidance, sub-cellular synaptic connectivity, and synapse maintenance. To control these diverse cellular processes, the Musashi proteins can fully rely on their versatility in terms of molecular function. Namely, Msi proteins can inhibit or stimulate mRNA translation, enhance polyadenylation, and regulate splicing (Sutherland et al., 2013; Fox et al., 2015; Murphy et al., 2016). In this section, we discuss how the versatility of Msi is exploited for neuronal wiring in different systems. First, we will introduce the well-described essential Msi functions in neuronal stem cell maintenance, cell proliferation, and cell fate determination. Subsequently, we will discuss Msi functions in neuronal morphology, axon guidance, synaptic connectivity and synapse maintenance.

Two homologues form the Msi protein family, which is conserved from invertebrates to vertebrates (Nakamura et al., 1994; Sakakibara et al., 1996, 2001; Nagata et al., 1999; Shibata et al., 2012). Msi proteins are highly enriched in the developing CNS, with prominent expression in embryonic, fetal, and adult neural stem cells (Nakamura et al., 1994; Sakakibara et al., 1996, 2001; Kaneko et al., 2000; Shibata et al., 2012). The name of the Msi protein was inspired by the samurai Miyamoto Musashi, who used to fight with two swords simultaneously: in *Drosophila*, where Msi was originally identified (Nakamura et al., 1994), disrupted asymmetric division of sensory organ precursors in *msi* null mutants leads to the duplication of large thoracic sensory bristles, and these duplicated bristles resemble the two swords that Musashi used (Nakamura et al., 1994).

Msi proteins contain two RNA binding domains (Sakakibara et al., 1996, 2001; Nagata et al., 1999; Ohyama et al., 2008; Iwaoka et al., 2017). Close to their C-terminus, an intrinsically disordered region can promote RNA binding (Iwaoka et al., 2017). Msi can bind to the pentamers-heptamers (G/A)U_1−3_AGU in RNA (Ohyama et al., 2012; Zearfoss et al., 2014; Schneider and Wolfinger, 2019). RNA-protein immunoprecipitation assays have uncovered more than 1,000 potential RNA targets of Msi (Vo et al., 2012; Uren et al., 2015; Bennett et al., 2016). This high number of targets explains the versatility of Msi function in different cellular processes.

### 3.1. Function of Msi Proteins in Neural Stem Cell Maintenance and Neuronal Proliferation

In the context of neural stem cell maintenance, cell proliferation, and cell fate determination, Msi functions by inhibiting translation of target mRNAs (Sutherland et al., 2013; Fox et al., 2015). Murine Musashi 1 (Msi1) is key to maintaining multipotent neuronal progenitors in the proliferative state, and it also influences cell differentiation (Sakakibara and Okano, 1997). Neuronal progenitors have high levels of Msi1, while differentiated neurons have lower Msi1 levels (Sakakibara and Okano, 1997). *In vitro* studies showed that Msi1 controls the proliferative state of neuronal stem cells *via* the cyclin-dependent kinase inhibitor p21^WAF-1^. In HEK293 cells, Msi1 binds to the 3′UTR of *p21*^*WAF-1*^ mRNA and represses its translation (Battelli et al., 2006). p21 is important to maintain cellular quiescence. Its regulation by murine Msi1 is a means to control quiescence vs. proliferation of (neural) stem cells (Qiu et al., 2004; Battelli et al., 2006). In the absence of murine *msi1*, the differentiation potential of neuronal precursors is lost (Sakakibara et al., 2002). *In vitro* studies showed that during cell differentiation, Msi1 modulates the Notch pathway by binding to the 3′UTR of *numb* mRNA, which results in inhibition of *numb* mRNA translation and thereby an increase in Notch signaling (Imai et al., 2001; Berdnik et al., 2002; das Chagas et al., 2020). Based on information collected from mammalian model systems, the mechanism proposed for this type of inhibition is mediated by Msi1 physically interacting with the Poly(A) binding protein (PABP), both bound to *numb* mRNA (Kawahara et al., 2008). Thus, Msi sequesters PABP and prevents its interaction with the eukaryotic translation initiation factor eIF4G. Reduced binding of eIF4G to PABP impedes the formation of the 80S ribosome and inhibits the initiation of translation (Kawahara et al., 2008).

As revealed by the study of Zika virus-induced microcephaly, the roles of Msi in stem cell maintenance, cell proliferation, and cell fate determination appear relevant to understanding the pathophysiology of this developmental disorder of the brain. The emergence of a Zika virus (ZIKV) epidemic in Brazil in 2016 showed that children that were exposed to the virus infection in the uterus developed defects ranging from mild developmental delay to severe microcephaly and other severe brain abnormalities (Kindhauser et al., 2016; Caldas-Garcia et al., 2020). Resulting from multiple efforts to understand the mechanisms of action of the virus, mammalian Msi1 was eventually shown to interact with the Zika genome (Chavali et al., 2017). The genomic RNA of the Brazilian ZIKV strain, PE243, has 3 Musashi binding elements (MBEs; Chavali et al., 2017). Msi1, but not Msi2, binds to the 3′UTR of the ZIKV and enhances ZIKV protein expression, which enables viral replication, at least in cultured neuronal cell lines (Chavali et al., 2017). The concomitant finding that Msi1 is mutated in individuals with autosomal recessive primary microcephaly suggested the following working model: because of binding of Msi1 to ZIKV RNA, ZIKV infection could induce microcephaly by titrating Msi1 protein. Thus, less Msi1 protein is available for binding to endogenous targets, leading to de-regulation of these endogenous targets during brain development (Chavali et al., 2017). This could lead to aberrant stem cell maintenance, cell proliferation, and cell fate determination in the CNS (Chavali et al., 2017). *In silico* studies showed that Msi1 can also bind to the 3′UTR of other, related flavoviruses (Schneider and Wolfinger, 2019). Therefore, several emerging viruses could cause the same developmental defects in children as the ZIKV (Schneider and Wolfinger, 2019).

Of note, Msi proteins have also been associated with neurodegenerative diseases. Msi proteins have intrinsically disordered regions that could lead to their aggregation and interaction with Tau (Chen and Huang, 2020; Montalbano et al., 2020), and Msi1/2 were found to form oligomers in brains of patients with Alzheimer's disease, amyotrophic lateral sclerosis, and frontotemporal dementia (Sengupta et al., 2018; Montalbano et al., 2020).

### 3.2. Functions of Msi Proteins in Neural Circuit Formation

**Figure 4:**
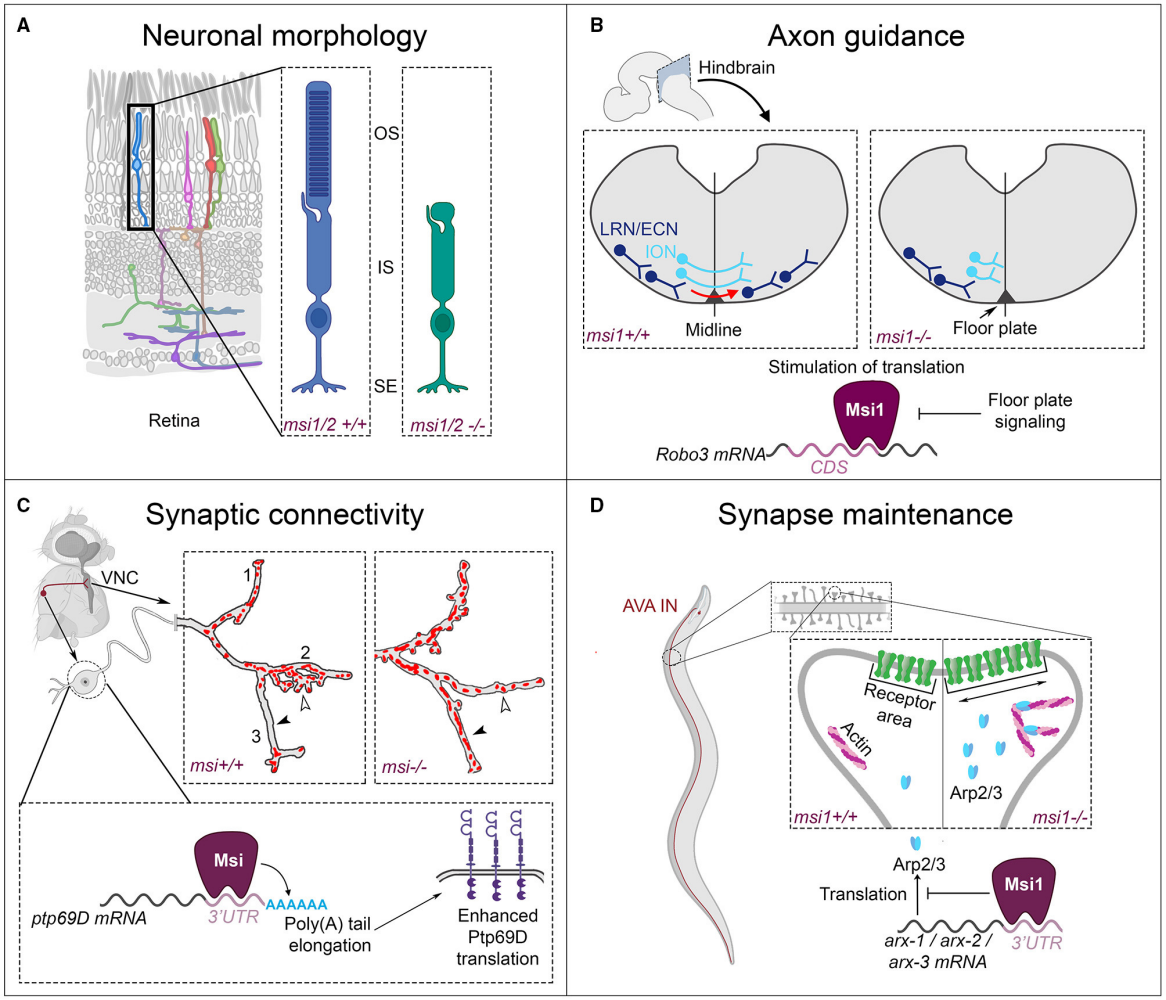
The RBP Musashi as a master regulator of neuronal wiring. **(A)** Msi proteins control the morphology of photoreceptors. In the mouse retina, Msi1 and Msi2 are required for the correct formation of the outer segment (OS) in photoreceptors. Moreover, in animals lacking Msi1/2, photoreceptors have impaired light response and increased degeneration. These phenotypes are attributed to dysregulation of a Msi1/2-dependent splicing program. IS, inner segment; SE, synaptic ending. Cartoon of retina reproduced from Baden, Tom (2020); Zebrafish retina (adult); Zenodo; https://doi.org/10.5281/zenodo.3926525. **(B)** Msi1 controls midline crossing of precerebellar neurons in mice. The precerebellar lateral reticular neurons (LRN) and external cuneate nucleous neurons (ECN) migrate toward the ventral midline and both the processes and the cell bodies cross the ventral midline. The inferior olivary neurons (ION) also migrate toward the ventral midline, but only their processes cross the midline. In animals lacking Msi1, midline crossing and neuronal migration of IO and LR/EC neurons are impaired. Msi1 binds to the coding sequence (CDS) of the *Robo3* mRNA to enhance protein levels of the Robo3 receptor. When the neurons are approaching the ventral midline, signaling from the floor plate inhibits Msi1 expression, which reduces *Robo3* translation. The temporal and spatial regulation of *Robo3* translation is required for the midline crossing of precerebellar neurons. **(C)** Msi controls axon collateral branch-specific synaptic connectivity of mechanosensory neurons in *Drosophila*. The mechanosensory neurons innervate bristles on the dorsal thorax of the fly and extend their axon to the ventral nerve cord (VNC). The axon forms three collateral branches that innervate the anterior (1), contralateral (2), and posterior (3) regions of the VNC, respectively. Msi specifically promotes the formation of terminal arborizations and a high number of synapses in branch 2 (open arrowhead). By contrast, in branch 3, Msi prevents ectopic synaptogenesis (filled arrowhead). These antagonistic, compartment-specific functions of Msi may depend on the regulation of different mRNA targets. For the function observed in branch 2, Msi binds to the 3′UTR of the mRNA encoding the receptor protein tyrosine phosphatase Ptp69D (bottom). Msi enhances poly(A) tailing and stability/translation of the *ptp69D mRNA*. For the function observed in branch 3, the relevant target(s) have not yet been identified. **(D)** Msi1 controls synapse size after associative learning in the *Caenorhabditis elegans* AVA interneuron. In wild-type animals, synapse/dendritic spine size increases during learning, and it is decreased *via* a Msi1-dependent mechanism during forgetting. Msi1 binds to the 3′UTR of transcripts coding for components of the actin branching regulator Arp2/3 to down-regulate its translation. The decrease in levels of the ARP2/3 complex leads to a reduced ramification of actin filaments, which correlates with the decrease in synapse size. In animals lacking Msi1, the translation of Arp2/3 complex components is not inhibited and synapse size remains high, leading to a failure to forget.

Surprisingly little is known about the roles of Msi in neural circuit formation beyond stem cell maintenance, cell cycle progression, and cell fate specification. Only few studies have investigated the functions of Msi in later steps of neuronal development that could contribute to the patterning of neuronal connectivity. Yet, it appears that Msi can regulate various aspects of postmitotic neuronal morphogenesis, axon guidance, the establishment of synaptic connectivity, and synapse maintenance (Figure 4), with dramatic impacts on neuronal wiring. Examples thereof are discussed below.

#### 3.2.1. Functions of Msi Proteins in Neuronal Morphogenesis

Murine Msi1/2 are involved in shaping the morphology of photoreceptors (Figure 4A): upon loss of Msi1/2, the outer segment of the photoreceptors is not formed (Sundar et al., 2020; Figure 4A). This phenotype seems to be due to the dysregulation of a specialized splicing program: Msi1/2 promote the inclusion of photoreceptor-specific exons in at least half a dozen transcripts that are critical for morphogenesis of the outer segment, and for synaptic transmission in mice and *X. laevis* (Murphy et al., 2016; Sundar et al., 2020). Remarkably, overexpression of Msi1 in liver cancer cells can induce the inclusion of photoreceptor-specific exons (Ling et al., 2020). However, the effect of Msi1/2 on AS in photoreceptors may be indirect: Msi1/2 could potentially have a broad impact on AS by regulating translation of RBPs that in turn regulate splicing. Finally, Msi1 is not a general regulator of splicing, but rather its function in splicing is restricted to specific cell types and/or specific genes. For example, in cellular models of glioblastoma, only the splicing of very few genes is controlled by Msi1 (Uren et al., 2015).

#### 3.2.2. Functions of Msi Proteins in Neuronal Migration and Axon Guidance

The evolutionary conserved Msi1 is also involved in regulating cell migration and axon guidance in mice. In pre-cerebellar neurons, this occurs *via* post-transcriptional regulation of the Robo3 cell-surface receptor (Kuwako et al., 2010; Figure 4B). Msi1 binds to the mRNA of *Robo3* at binding elements in the coding sequence, in contrast to the vast majority of confirmed Msi1 targets, for which binding occurs in the 3′UTR. Interestingly, *Robo3* binding is not mediated by consensus MBEs, but by alternative elements that are unknown (Kuwako et al., 2010). Loss of *msi1* leads to a decrease in Robo3 protein levels, without affecting the *Robo3* mRNA levels (Kuwako et al., 2010), suggesting that Msi1 directly stimulates *Robo3* translation. Very similar neuronal migration and axon guidance phenotypes are observed in precerebellar neurons of animals either lacking *Robo3* or *msi1* (Kuwako et al., 2010). Moreover, it is worth noting that Msi1 was found to positively regulate YTHDF1 expression in glioblastoma (Yarmishyn et al., 2020). Since YTHDF1 positively regulates Robo3 expressions in spinal commissural neurons (see above), it will be an interesting avenue for future studies to determine if YTHDF1 regulation is a second, parallel Msi1-dependent or -independent pathway to control Robo3 expression in developing neurons.

#### 3.2.3. Functions of Msi Proteins in Establishing Synaptic Connectivity

In the *Drosophila* CNS, we recently identified a role for Msi in the sub-cellular control of synaptic connectivity. Msi specifically promotes a high number of synapses in one axon collateral of mechanosensory neurons, while in a different compartment of the same axon, Msi limits synapse number and prevents ectopic synaptogenesis. Thus, Msi has opposing, compartment-specific functions (Landínez-Macías et al., 2021; Figure 4C). Moreover, Msi has an additional function in promoting the formation/growth of a specific axon collateral branch. Msi binds to the 3′UTR of the mRNA encoding the type IIA receptor protein tyrosine phosphatase Ptp69D and enhances its poly(A) tailing (Landínez-Macías et al., 2021). Our study proposes that the regulation of polyadenylation is a means to control translation of the *ptp69D* mRNA. In turn, precisely set levels of Ptp69D protein determine synaptic connectivity in one specific axon compartment (Landínez-Macías et al., 2021). The different compartment-specific functions downstream of an RBP present a concept of how a single “master” regulator can confer subcellular specificity of morphogenesis (in this case, synaptogenesis), thereby reducing the complexity required for the regulation of gene expression. Molecularly, we propose that Msi regulates different mRNA targets in mechanosensory neurons, which, in turn, have opposing functions in the control of synapse numbers in different subcellular compartments. As previously described for non-neuronal cells (Arumugam et al., 2012; Cragle and MacNicol, 2014; Weill et al., 2017), our findings further support that Msi proteins can not only function in the inhibition of translation but can also be translational activators for specific target mRNAs. How is this activation achieved on the molecular level? Different mechanisms were identified by studies in *X. laevis* oocytes. Msi1 induces oocyte maturation by activating translation of target mRNAs at specific time points during meiotic progressions (Arumugam et al., 2012). This translational activation can be achieved by stimulation of polyadenylation. In one scenario, the binding of Msi1 to the 3′UTR of target mRNAs can elicit structural changes that lead to preferential exposure of adjacent cytoplasmic polyadenylation elements (CPEs), and induction of polyadenylation (Cragle and MacNicol, 2014; Weill et al., 2017). CPEs are recognized by the cytoplasmic polyadenylation element binding protein (CPEB), which on the one hand dictates, which mRNAs undergo cytoplasmic polyadenylation, and on the other hand impacts on the strength of polyadenylation. In another scenario, *via* a mechanism independent of CPEB, Msi1 can associate with Gld2 (germline development 2), a protein that catalyzes poly(A) addition (Cragle and MacNicol, 2014). Msi1 bound to Gld2 directs cytoplasmic polyadenylation and activation of translation of Msi targets (Cragle and MacNicol, 2014). These mechanisms could be conserved in the mammalian brain: CPEB and Gld2 were shown to elicit polyadenylation of neuronal mRNAs in mouse hippocampal neurons (Zearfoss et al., 2008; Udagawa et al., 2012). However, it remains to be tested if in neuronal tissues Msi1 also interacts with Gld2 or CPEB to enhance polyadenylation of specific targets. Surprisingly, while Msi1 can *inhibit* translation by binding to PABP and thus decrease PABP interactions with the translation initiation complex (see above), Msi1-mediated translational *activation* in *X. laevis* oocytes can also be achieved *via* an interaction with an embryonic PABP, or the canonical somatic cell PABPC1 (Cragle and MacNicol, 2014).

In general, it remains largely unknown how RBPs shape neuronal circuits through the specific control of poly(A) tailing. Besides the cytoplasmic polyadenylation element binding protein and other general regulators of polyadenylation, only a few RBPs with functions linked to the poly(A) tail have been involved in neuronal wiring. One of them is the Nab2 poly(A) binding protein, which controls the guidance of *Drosophila* mushroom body (MB) axons (Bienkowski et al., 2017). Thus, the control of mRNA polyadenylation is an underappreciated mechanism for the translational regulation of specific mRNAs in developing neurons, and an interesting avenue for future studies.

#### 3.2.4. Function of Msi Proteins in Synapse Maintenance

In *C. elegans*, Msi has been linked to the control of synapse size and of time-dependent memory loss (Figure 4D). Msi1 (the only Msi paralog in *C. elegans*) binds to the 3′UTR of the mRNAs encoding three different subunits of the Arp2/3 complex (Hadziselimovic et al., 2014). Thereby, Msi1 elicits downregulation of Arp2/3 expression. This mechanism causes the reversal of learning-induced increases in synapse size. Hence, synapse size cannot be reduced in animals lacking *msi1*, which leads to deficits in forgetting. These findings thus link the translational control of cytoskeletal components to forgetting (Hadziselimovic et al., 2014). Forgetting is an essential physiological process whose regulation/dysregulation is at the core of neurological syndromes such as post-traumatic stress disorder or the extremely rare Hyperthymestic Syndrome/Highly Superior Autobiographical Memory (Parker et al., 2006; LePort et al., 2012). Interestingly, the most prominent targets of the Msi family of proteins, identified by transcriptome-wide mapping of RNA targets, are key regulators of the actin cytoskeleton and of focal adhesions (Vo et al., 2012; Uren et al., 2015; Bennett et al., 2016; Lin et al., 2019). In particular, Filamin, Rac, WAVE1, α-catenin, and actin can be bound by Msi (Uren et al., 2015). These proteins have key roles in axon outgrowth, branching and targeting, and synapse formation and maintenance, and are interesting putative targets to further study Msi-mediated mechanisms that control neuronal wiring.

## 4. Malfunction of RNA Binding Proteins and Associated Diseases

Neurodevelopmental and psychiatric disorders affect a considerable proportion of the population worldwide, with prevalences of several percent for certain syndromes, and they are highly driven by genetic determinants (Parenti et al., 2020). Yet, our understanding of the molecular etiology of these disorders is still very limited. Risk loci identified in genome wide association studies have paved the way for investigating these underlying molecular mechanisms. As illustrated so far in this article, gene expression in the central nervous system is highly regulated during development and in a tissue and cell type-specific manner and this control is essential for the formation of complex neural circuits. Thus, not surprisingly, an important number of risk loci for neurodevelopmental and psychiatric disorders are present in non-coding and regulatory regions of the genome (Schizophrenia Working Group of the Psychiatric Genomics Consortium, 2014; Parikshak et al., 2016). In particular, genetic variants in RBP binding sites (required for RBP-mediated post-transcriptional regulation of gene expression) are drivers of psychiatric disorder risk, with, remarkably, an even stronger impact than genetic variants in coding regions (Park et al., 2021). In this section, we exemplify how dysregulation of RBP-RNA interactions, at several steps of RNA metabolism, can lead to disease. We will highlight the molecular and cellular functions of some RBPs and how their loss/reduction of function can lead to disease. Given that RBPs can have thousands of targets, we will specially emphasize non-monogenic disorders. Several “infamous” RBPs, such as TDP-43, FUS, FMR1/FMRP, and SMN, are causally linked to different neurological disorders. The function of these proteins has been extensively reviewed elsewhere (Hagerman et al., 2018; Bagni and Zukin, 2019; Gao et al., 2019; Prasad et al., 2019; Wirth et al., 2020; Zbinden et al., 2020; Portz et al., 2021) and although many questions regarding the cellular and molecular consequences of their dysregulation remain open, we will not discuss them here.

### 4.1. Alternative Splicing

Genetic variants in loci linked to splicing have a big impact on the risk for disorders of brain development (Parikshak et al., 2016; Walker et al., 2019), and altered function of splicing factors can lead to neurodevelopmental disorders, such as intellectual disability (ID; Mattioli et al., 2020). For example, a point mutation in *Nova2* that is found in patients with ID leads to a reading frame shift that changes the C-terminal part of the protein (Mattioli et al., 2020). This mutation impairs Nova2 RNA binding activity and causes dysregulation of AS events, with, among the affected targets, an enrichment of mRNAs coding for regulators of the cytoskeleton. The C-terminal frame shift also elicits aberrant axon tract formation *in vivo* in the zebrafish visual system, suggesting that axonal wiring defects may underlie the human neurodevelopmental disorders caused by mutations in *Nova2* (Mattioli et al., 2020).

An intriguing feature of gene expression in the nervous system is the alternative use of microexons (3-27 nt long). These microexons are frequently neuron-specific, and generate in-frame changes in the coding sequence, which lead to variant protein surfaces and modulation of protein-protein interactions (Irimia et al., 2014). Dysregulation of microexon splicing is present in many disease-associated proteins, including proteins linked to autism spectrum disorders (ASD; Irimia et al., 2014). One of the key regulators of inclusion of microexons in neuronal cells that has been linked to ASD is Srrm4 (also known as nSR100; Irimia et al., 2014; Quesnel-Vallières et al., 2015; Gonatopoulos-Pournatzis et al., 2018). Quesnel-Vallières et al. developed a mouse model of ASD with a reduction of Srrm4 expression to 50% of wild-type levels, in order to study the molecular functions of this splicing regulator. They found that reduction of Srrm4 protein levels leads to deficits in social behavior as observed in ASD (Quesnel-Vallières et al., 2016). Also, the 50% reduction of Srrm4 is sufficient to alter the splicing of microexons in targets of Srrm4 that are associated with ASD, including cues and receptors involved in axon guidance and synaptic maturation/transmission, such as Slit2, Dnm2, and Nrxn2 (Quesnel-Vallières et al., 2016). The impact of these changes on the neural circuitry was demonstrated in pyramidal neurons of the somatosensory cortex: upon reduction of Srrm4 levels, these neurons had a strong reduction in synaptic transmission and neuronal excitability. At the morphological level, only minor changes were observed upon 50% reduction of Srrm4 levels, namely in the morphology of dendritic spines (Quesnel-Vallières et al., 2016). By contrast, full knockout of Srrm4 leads to severe wiring defects, such as impairment of neurite outgrowth in motor neurons, disruption of cortical layering, premature neurogenesis, and midline crossing defects (Quesnel-Vallières et al., 2015).

Mechanistically, Srrm4 regulates splicing of microexons in association with the SR-related proteins Srsf11 and Rnps1 (Gonatopoulos-Pournatzis et al., 2018). *In vitro* studies showed that Srrm4, Rnps1, and Srsf11 form a specialized splicing enhancer complex that binds to intronic sequences upstream of neuronal microexons and promote early steps in spliceosome assembly (Gonatopoulos-Pournatzis et al., 2018). This complex regulates several microexons that are affected in patients with ASD (Gonatopoulos-Pournatzis et al., 2018), including a specific microexon in the mRNA encoding the translation initiation factor eIF4G1 (Gonatopoulos-Pournatzis et al., 2020). Recapitulating defects observed in ASD, lack of inclusion of this microexon in a mouse model leads to impaired social behavior and abnormal protein synthesis-dependent synaptic plasticity (Gonatopoulos-Pournatzis et al., 2020). Molecularly, in the same mouse model, the steady-state levels of several proteins with key synaptic functions are increased upon exclusion of the microexon in eIF4G1, such as a subunit of NMDA-type ionotropic glutamate receptors, and the postsynaptic components of inhibitory synapses Gephyrin and Neuroligin-2 (Gonatopoulos-Pournatzis et al., 2020). Inclusion of the microexon in *eIF4G1* increases the propensity of eIF4G to undergo phase separation, which allows the formation of neuronal RNP granules with Fxr1, Ataxin-2, Larp1, and Stau2, and causes ribosome stalling (Gonatopoulos-Pournatzis et al., 2020). Hence, the absence of the microexon in *Eif4g1* mRNA leads to enhanced translation of synaptic proteins and alterations in synaptic transmission (Gonatopoulos-Pournatzis et al., 2020). Remarkably, another general regulator of translation that is associated with ASD risk and regulated by Srrm4/Srrm3/Srsf11-dependent microexon splicing is the cytoplasmic polyadenylation element binding protein 4 (CPEB4; Quesnel-Vallières et al., 2015; Parras et al., 2018). CPEB4 AS is altered in patients with ASD, with a decrease of microexon 4 inclusion and a reduction of protein levels (Parras et al., 2018). In a mouse model recapitulating *CPEB4* microexon 4 skipping, deadenylation is enhanced and translation is reduced for mRNAs of ASD risk genes (Parras et al., 2018). These mice also show a decreased spine density in dendrites of pyramidal neurons in the somatosensory cortex, and behavioral changes recapitulating the hallmarks of ASD (Parras et al., 2018). It is not clear if the eIF4G and CPEB4 proteins converge to control the translation of the same mRNA targets, and the formation of neuronal granules in the same cell types/developmental stages. However, insights into the (dys)regulation of eIF4G, CPEB4, and the Srrm4/Srrm3/Srsf11 complex together could strengthen our understanding of the root causes of ASD, and provide an entry point for tackling the disorder.

### 4.2. Regulation of Translation

RNA-binding proteins control the translation of a plethora of targets at different stages in the process, namely the initiation, elongation, or termination of translation, or ribosome recycling (Sonenberg and Hinnebusch, 2009). Not surprisingly, mutations altering the function and/or expression levels of RBPs with functions in translational regulation can be at the core of different neurodevelopmental disorders and also elicit accompanying syndromes. For example, a mutation that affects the C-terminal domain of the eukaryotic translation initiation factor eIF2γ was identified as a possible cause of MEHMO syndrome (Young-Baird et al., 2020). This syndrome is characterized by intellectual disability (ID), epilepsy, hypogenitalism, microcephaly, and obesity. In patient-derived induced pluripotent stem cells, the eIF2γ mutation decreases global translation levels due to defective assembly of eIF2 complexes (Young-Baird et al., 2020), which under normal circumstances assist in the selection of the translation initiation site and the delivery of the initiation methionyl-tRNA (Hinnebusch, 2014).

As another example, DDX3X is an RNA helicase of the DEAD-box family that is associated with ASD, amyotrophic lateral sclerosis, frontotemporal dementia (Cheng et al., 2019), and Toriello Carey (Lennox et al., 2020). Toriello Carey is a disease characterized by ID, corpus callosum agenesis, facial malformations, abnormal postnatal brain development leading to microcephaly, respiratory distress, and cardiovascular anomalies (Toriello et al., 2016). DDX3X is involved in the regulation of canonical translation, repeat-associated non-AUG translation, and formation of RNP granules (Cheng et al., 2019; Hondele et al., 2019; Lennox et al., 2020). Lennox et al. (2020) generated a mouse model with the DDX3X mutations found in patients with ID, which reduce DDX3X helicase activity. In this model, the degree of impairment in the helicase activity correlates with the severity of the symptoms found in patients. Cellularly, reduced DDX3X helicase activity leads to a decrease in cortical neuron generation and in neuronal differentiation during mouse prenatal development. Molecularly, the disruption of DDX3X helicase activity alters the translation of some mRNA targets and elicits ectopic RNP granules in neuronal progenitors (Lennox et al., 2020). In addition, in these mutants, the translation of proteins with repeat-associated non-AUG translation fails to be repressed, which leads to the accumulation of cytotoxic dipeptide repeat proteins (Cheng et al., 2019).

Other RBPs associated with complex neurological disease phenotypes have more specific effects in regulating a smaller group of genes. For example, mutations in Cold Shock Domain-containing E1 (CSDE1) are associated with ASD and ID (Xia et al., 2014; Guo et al., 2019). This RBP was initially studied in the context of stem cell maintenance and the inhibition of neuronal differentiation (Ju Lee et al., 2017). Similarly as our previous example Msi, roles for CSDE1 in later steps of neuronal development have emerged more recently. Downregulation of *CSDE1* in mouse cortical pyramidal neurons leads to neurite overgrowth and reduced branching, decreased spine density, immature spine morphology, reduced number of synapses, and defects in synaptic transmission (Guo et al., 2019). The molecular mechanism mediating these defects is proposed to be the dysregulation of components of the Wnt/β-catenin cell adhesion pathway, including β-catenin, APCDD1, and CDH2 (Guo et al., 2019; El Khouri et al., 2021). Remarkably, expression of β-catenin is sufficient to rescue the developmental defects observed upon downregulation of *CSDE1* (Guo et al., 2019). The function of CSD1 in synapse development is conserved for Unr (the *Drosophila* homolog of mammalian CSD1; Guo et al., 2019).

The transcription factor Ataxin-1 is a good example underscoring the importance of precise regulation of protein levels. Changes in Ataxin1 levels cause spinocerebellar ataxia type 1 (SCA1), which is characterized by cerebellar degeneration due to the accumulation of the protein Ataxin1. Mutated Ataxin1 can contain expanded polyglutamine repeats, which protect the protein from degradation by the proteasome and thus lead to higher protein levels in mice models of SCA1 (Cummings et al., 1999). Increased Ataxin1 levels can also arise from insufficient translational repression. The Pumilio1 RBP binds to the 3′UTR of *Ataxin1* mRNA, and, under normal conditions, decreases the stability of the mRNA, which reduces both mRNA and protein levels of Ataxin1 (Gennarino et al., 2015). In mice models of SCA1, reduction of Pumilio1 protein levels can lead to SCA1 due to overexpression of wild-type Ataxin1, a phenotype that can be rescued by decreasing Ataxin1 levels. However, additional neurological features in Pumilio1 mutants cannot be rescued by this manipulation, suggesting additional pathways (Gennarino et al., 2015). Interestingly, human *Pumilio1* mutations that differentially reduce its protein levels lead to a different time of disease onset, with lower Pumilio1 levels correlating with earlier onset time and stronger symptoms in patients. A 50% reduction in Pumilio1 causes developmental delay and ID in addition to ataxia (Gennarino et al., 2018), highlighting the developmental roles of Pumilio1 in addition to its roles in the maintenance of neuronal circuits.

RBPs, such as FMRP, Ataxin-2, and SMN (linked to the neurological diseases fragile X mental retardation, spinocerebellar ataxia type 2, and spinal muscular atrophy, respectively) are implicated in the control of mRNA localization and local translation in neurons (Dictenberg et al., 2008; Akten et al., 2011; Fallini et al., 2011; Sudhakaran et al., 2014). The precise causal relationships between defects in mRNA localization/local translation and neurological diseases are however only beginning to be unraveled. As these advances have been discussed in two recent reviews (Wang et al., 2016; Thelen and Kye, 2019), we will not further elaborate on them here.

### 4.3. RNA Editing

Impairment of ADAR-mediated RNA editing activity has been associated with different neurological disorders, including ALS (Aizawa et al., 2010; Hideyama et al., 2012), epilepsy (Srivastava et al., 2017), Alzheimer's disease (Khermesh et al., 2016), autoimmune disorders (Rice et al., 2012), and ASD (Eran et al., 2013; Tran et al., 2019). Interestingly, in mammals, ADAR expression can be stimulated by interferon (IFN) activity (Patterson and Samuel, 1995), and at the same time, ADAR can suppress IFN signaling (Hartner et al., 2009), suggesting a negative feedback loop. In a mouse model of viral infection that recapitulates ASD-associated behavioral changes, ADAR activity is increased, possibly due to IFN-induced ADAR expression (Tsivion-Visbord et al., 2020). As a consequence, the levels of RNA editing are increased in transcripts coding for proteins involved in neuronal development, metabolism, and the immune response. *FLN-*α and *FLN-*β were among the identified ADAR targets with altered levels of editing (Tsivion-Visbord et al., 2020). As discussed before, an interesting model is that dysregulation of editing in *FLN-*α and *FLN-*β could lead to changes in neuronal wiring, which in turn could be the cause of the observed behavioral changes.

Mutations in ADAR have been found in patients with Aicardi-Goutéres syndrome (Rice et al., 2012), an autoimmune disease characterized by brain inflammation due to increased IFN signaling (Crow and Livingston, 2008). In these patients, the mutations found in ADAR can lead to a decrease of its catalytic activity (Rice et al., 2012). Aicardi-Goutéres syndrome patients have an increase in expression of IFN stimulated genes. However, the authors discuss that it is not clear whether loss of ADAR catalytic activity is the cause of increased IFN signaling (Rice et al., 2012). Can the inflammation elicited by increased IFN signaling induce neuronal wiring defects? This is just one of the many open questions regarding the role of RNA editing, and the underlying cellular and molecular principles, in patients with neurodevelopmental and psychiatric disorders.

Changes in ADAR activity are found in individuals with ASD, also independently of IFN signaling. A postmortem study comparing the editing activity in cerebellar tissue of individuals diagnosed with ASD to the editing activity in individuals without diagnosis showed differences in editing of proteins involved in synaptic transmission (Eran et al., 2013). Interestingly, alterations could go into either direction: for the same editing site, a percentage of editing higher or lower than the average values found in undiagnosed individuals were found in samples from individuals with ASD (Eran et al., 2013). The observed changes might be due in part to the altered expression of ADAR (Eran et al., 2013). In ASD samples, an ADAR isoform with defective binding to dsRNA is expressed at higher levels, and simultaneously, there is downregulation of the ADAR isoform that can actually bind dsRNA (Eran et al., 2013). The study also found that altered RNA editing is correlated with alternative exon retention, affecting *FLN-*α, among others. More specifically, editing at the 3′ end of exon 43 in *FLN-*α mRNA leads to higher exon retention (Eran et al., 2013). It remains to be elucidated if and how increased variability in the editing of *FLN-*α, and therefore increased variability in exon 43 retention, altogether affect neuronal wiring. A second study, with a larger cohort of patients, studied the editing patterns in the frontal cortex, temporal cortex, and cerebellum (Tran et al., 2019). This study also found that ASD samples had higher variability of editing, although there was a higher proportion of sites with lower editing rates (Tran et al., 2019). The genes with larger changes in editings have functions in synaptic transmission, corroborating previous studies describing ADAR function. Several of these editing sites belong to genes that are regulated by FMRP and FXR1P (Tran et al., 2019). The ADAR-edited sites are found in close proximity to the binding sites of these RBPs, and both, FMR1 and FXR1P function regulate the editing of these sites. Remarkably, samples from Fragile X patients, an ID with phenotypes overlapping ASD, showed similar changes in RNA editing as ASD sample (Tran et al., 2019).

These studies provide an entry point to understand the role of ADAR in neurodevelopmental diseases. However, more efforts are needed to further characterize other proteins that interact with ADAR and that modulate its activity. Also, it is required to dissect how changes in RNA editing of ADAR targets affect their cellular and molecular functions, and if and how this could modify neuronal wiring.

## 5. Conclusions and Future Perspectives

Wiring of the nervous system, with several 100,000 neurons (*Drosophila*) to billions of neurons (86 billions for humans, 200 billions for elephants) is a daunting task. Division of the process into temporally and spatially separated, successive steps, and the application of “simple” pattern formation rules, reduces the complexity that needs to be encoded in the genome (Hassan and Hiesinger, 2015). This breaking down of neural circuit formation into several steps requires precise, localized control of gene expression, often on a timescale of minutes. As we have discussed, local translation in neuronal processes has emerged as a crucial component of this regulation. Thereby, neurons need to rapidly integrate extrinsic and intrinsic cues. While clear *in vivo* evidence for local translation has now been provided for dendrites, (developing) axons and both, pre- and postsynapses, much about its extent and functions in developmental processes, such as axon branching and targeting, remains elusive. A large body of knowledge on local translation in the axon is derived from studies of cultured cells, or of bulk isolates of axons from brain tissue. Studies of single, defined cells (/cell types) and even subcellular compartments *in vivo* will be required to tackle questions such as: how do neurons integrate many extrinsic and intrinsic cues in a rapid way? How are the global and the subcellular proteome changing in response to the specific set of cues that an axon/growth cone is exposed to? Why do different axons respond differently? What are the molecular transduction mechanisms, i.e., how does the (in)activation of a particular cell-surface receptor lead to increases or decreases in global translation, or of only a specific set of RNAs? What is the role of RBPs in this context, do they act as all-or-nothing “molecular switches,” or rather in dose-dependent gradual changes in the response? The latter is a major question not only in the context of local translation, but generally in the post-transcriptional control of gene expression.

Despite the mechanisms for reducing the complexity of nervous system wiring and thus of the genomic coding potential required for it, proteome expansion at the post-transcriptional level is essential for correct nervous system development. Except for select genes, some of which we have discussed here, the molecular underpinnings and roles of AS and APA, and their potential coupling (i.e., specific exons being linked to specific 3′UTR isoforms), remain unexplored for many genes and many neurodevelopmental processes. The same applies to other post-transcriptional events. For example, how much RNA editing is present in axons? Can RNA editing even be specific for subcellular compartments? Does this depend on levels of the Adar enzyme, or the localization of specific modulators of its activity? Or could edited RNAs be recognized and transported selectively to specific places in the cell? Likewise, the coupling between mRNA poly(A) tail length and protein production, which was found to differ between different developmental stages/cell types (Xiang and Bartel, 2021), remains unexplored for different neuronal subcellular compartments and wiring stages. Finally, a major question in our opinion is which RNA degradation pathways, besides the NMD pathway described above for control of Robo3.2 expression, are present in axons and dendrites and how they contribute to neuronal morphogenesis and connectivity. The tackling of these and other questions will be greatly facilitated by recent technological advances for the study of RNA metabolism, such as tools to visualize local translation *in situ*, and improved high-throughput sequencing approaches that can also be applied for epitranscriptomics, i.e., the study of RNA modifications. We will need to apply these techniques to study post-transcriptional events not only individually, but also how they are coupled and to what extent there is crosstalk between them. Such studies will provide major insights into how RNA metabolism shapes the nervous system under both, physiological and pathophysiological conditions.

## Author Contributions

ML-M and OU wrote, discussed, and edited the manuscript. All authors contributed to the article and approved the submitted version.

## Funding

This work was supported by grants from the Swiss National Science Foundation (SNSF; Ambizione PZ00P3_161448), from the Promotor Stiftung and from the Julius Klaus Stiftung to OU, and by funding from the University of Zurich.

## Conflict of Interest

The authors declare that the research was conducted in the absence of any commercial or financial relationships that could be construed as a potential conflict of interest.

## Publisher's Note

All claims expressed in this article are solely those of the authors and do not necessarily represent those of their affiliated organizations, or those of the publisher, the editors and the reviewers. Any product that may be evaluated in this article, or claim that may be made by its manufacturer, is not guaranteed or endorsed by the publisher.
